# A Mathematical Modelling Study of Chemotactic Dynamics in Cell Cultures: The Impact of Spatio-temporal Heterogeneity

**DOI:** 10.1007/s11538-023-01194-9

**Published:** 2023-09-08

**Authors:** Jacobo Ayensa-Jiménez, Mohamed H. Doweidar, Manuel Doblaré, Eamonn A. Gaffney

**Affiliations:** 1 Aragon Institute of Engineering Research, University of Zaragoza, Mariano Esquillor, s.n., 50018 Zaragoza, Spain; 2 Mechanical Engineering Department, School of Engineering and Architecture (EINA), University of Zaragoza, María de Luna s.n., 50018 Zaragoza, Spain; 3 Tissue Microenvironment Laboratory (TME Lab), Institute for Health Research Aragón, San Juan Bosco, 13, 50009 Zaragoza, Spain; 4 Wolfson Centre for Mathematical Biology, Mathematical Institute, University of Oxford, Woodstock Road, Oxford, OX2 6GG UK; 5 Nanjing Tech University, 30 South Puzhu Road, 211816 Nanjing, China

**Keywords:** Cell culture, Heterogeneous stimuli, Method of characteristics, Perturbation theory, Glioblastoma cell progression, Microfluidics

## Abstract

As motivated by studies of cellular motility driven by spatiotemporal chemotactic gradients in microdevices, we develop a framework for constructing approximate analytical solutions for the location, speed and cellular densities for cell chemotaxis waves in heterogeneous fields of chemoattractant from the underlying partial differential equation models. In particular, such chemotactic waves are not in general translationally invariant travelling waves, but possess a spatial variation that evolves in time, and may even oscillate back and forth in time, according to the details of the chemotactic gradients. The analytical framework exploits the observation that unbiased cellular diffusive flux is typically small compared to chemotactic fluxes and is first developed and validated for a range of exemplar scenarios. The framework is subsequently applied to more complex models considering the chemoattractant dynamics under more general settings, potentially including those of relevance for representing pathophysiology scenarios in microdevice studies. In particular, even though solutions cannot be constructed in all cases, a wide variety of scenarios can be considered analytically, firstly providing global insight into the important mechanisms and features of cell motility in complex spatiotemporal fields of chemoattractant. Such analytical solutions also provide a means of rapid evaluation of model predictions, with the prospect of application in computationally demanding investigations relating theoretical models and experimental observation, such as Bayesian parameter estimation.

## Introduction

Biological mechanisms require the orchestration of diverse cell populations, properties of the extracellular matrix (ECM), chemotactic gradients, and physical signals, to effect a complex, dynamic, and interactive microenvironment (Chen et al. 1997; Curtis and Seehar 1978; Schwarz and Bischofs 2005; Carter 1967; Lo et al. 2000). Cells continuously adapt to their surroundings, particularly to maintain homeostasis, keeping the intracellular and extracellular environment within physiological bounds (Bray 2000). In particular, in response to external stimuli, cells adapt, modifying numerous aspects of their behaviour, such as proliferation, gene expression, ECM production, migration and differentiation. Furthermore, these cellular responses impact the surrounding medium and neighbouring cells (Mousavi et al. 2013b; Kumar et al. 2013; Mousavi et al. 2014). This coupled interaction between cells and their environment is fundamental in developmental and physiological processes such as embryogenesis, organ development and tissue repair, as well as pathophysiologies such as atherosclerosis or cancer (Huang and Ingber 2005; Hanahan 2022; Nagelkerke et al. 2015; Quail and Joyce 2013; Urdeitx et al. 2023). Hence, advancing innovative frameworks to enhance our understanding of these mechanisms, together with their interactions, is essential for developing novel therapeutic strategies driven by promoting or inhibiting specific cellular behaviours (Mousavi et al. 2013a).

However, with in vivo research it is difficult to control and isolate effects, justifying in vitro research as an alternative to experimental medicine and studies with animals. Nonetheless, biological mechanisms proceed in three-dimensional structures (Edmondson et al. 2014), but in vitro cells are mostly cultured in a traditional Petri dish (2D culture). Indeed, the continuous drop in the number of drugs that reach the market, despite billion-dollar investments, demonstrates that the current predictive power of both in vivo and in vitro research still requires improvement (Scannell et al. 2012).

Recently, microfluidics has arisen as a powerful tool to recreate the complex microenvironment that governs tumour dynamics (Sackmann et al. 2014; Bhatia and Ingber 2014). This technique allows the reproduction of numerous important features that are lost in 2D cultures, as well as testing drugs in a much more reliable and efficient way (Bersini et al. 2014; Boussommier-Calleja et al. 2016; Jeon et al. 2015; Zervantonakis et al. 2012; Wu and Yotnda 2011).

In addition to microfluidic in vitro models, theoretical cell population evolution models based on transport partial differential equations (PDEs) have been increasingly used to investigate cells and their environmental interaction in diverse areas, as exemplified by cancer modelling (Byrne 2010; Altrock et al. 2015). A particular niche of interest is Glioblastoma (GBM), the most common and aggressive primary brain tumour (Brat 2012), with extensive studies dedicated to mathematical modelling its evolution (Hatzikirou et al. 2005), reproducing aspects of GBM histopathology (Bearer et al. 2009) and incorporating the influence of tumour microenvironment (TME) chemical and mechanical cues (Kim et al. 2016). It has been demonstrated that GBM progression is extensively controlled by the local oxygen concentrations and gradients (Hatzikirou et al. 2012), motivating many studies to incorporate the role of oxygen gradients and hypoxia in tumour progression (Ayuso et al. 2017; Martínez-González et al. 2012; Frieboes et al. 2006).

In particular, there has been a confluence of theoretical mechanism-based modelling and microfluidic experimental studies in the investigation of GBM cultures (Ayensa-Jiménez et al. 2020), where a *go-or-grow* transition switch, governed by nonlinear activation functions for the chemotaxis and growth in a modelling framework has reproduced GBM culture evolution under differing conditions. Such studies not only implicate the balance between cell chemotaxis and proliferative activity, and their relation to the different TME stimuli, as playing key roles in GBM evolution, but also emphasise how modelling can contribute to advances in microfluidic cell culture studies. However, numerically solving the model equations incurs a high computational cost in these applications, especially in the resolution of inverse problems such as parameter estimation, model selection, the design of experiments, sensitivity studies, model structural analysis and Uncertainty Quantification (UQ). Consequently, analytical solutions, even approximate, provide key information to test and validate numerical algorithms, inform a mechanism-based understanding across parameter space and to allow initial predictions for behaviours of interest, such as travelling fronts and equilibria, noting GBM progression has been considered in this manner (Pérez-García et al. 2011; Gerlee and Nelander 2016; Stepien et al. 2018).

Thus our objective is to develop rational analytical approximations in the modelling of the chemotactic cell motility dynamics, including situations of relevance for microfluidic studies of GBM cultures (Shin et al. 2012; Sackmann et al. 2014). In particular chemotaxis is typically represented via the framework of Keller and Segal (1971), which is well-known for its rich structure (Arumugam and Tyagi 2021; Xue et al. 2011). For many situations of interest for microdevice cell cultures, the problem may be considered one-dimensional and the chemotactic agent concentration, while heterogeneous, may be taken to be known, either because it can be directly measured, or because its concentration can be computed by solving a diffusion problem that is, to good approximation, decoupled from the cell population field. Consequently, our objective reduces to determining approximate analytical solutions for the one-dimensional chemotactic cell fronts in heterogeneous gradients of chemoattractants, for instance considering the time dependence in spreading speeds and impact of temporal oscillations of chemotactic stimuli. Of particular note, our study moves beyond traditional travelling wave analysis to analyse cell chemotactic invasive dynamics where the wavefront and wavespeed evolve in time.

To proceed, we first describe the structure of the one-dimensional mathematical problem associated with the response of cell populations to chemotactic gradients, together with the general assumptions and hypotheses about the underlying mechanisms. We derive pertinent features of the solutions, for instance migratory structures with a transition zone wavefront, with estimates of the time-dependent wavespeed and the shape of the solution profile. In particular, we compute an analytical solution for specific exemplar cases associated with specific relevant experimental situations, such as a constant spatial gradient of chemoattractant, temporal oscillations due to a fluctuating source and an exponential profile of chemoattractants corresponding to the diffusion from a localised source. We further apply the general results to the analysis of potential cell culture microfluidic experiments, representing a simplified version for an in vitro model of glioblastoma progression, developed by Ayensa-Jiménez et al. (2020), showing the potential for the methods presented here to generate analytical results for the simulation of microdevice representations for migratory tumour cell dynamics.

## Methods

### The Model

**Fig. 1:**
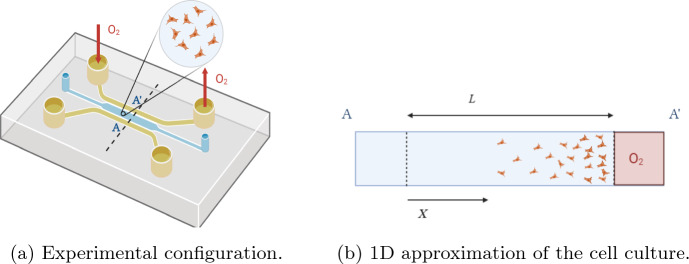
(Color Figure Online) Typical experimental configuration for modelling cell cultures. Due to the much larger length of the lateral channels relative to the width of the chamber, the domain geometry of the model is assumed one-dimensional, with axis *X* and width *L*, as illustrated. Using $t,~x=X/L$ to respectively denote non-dimensional time and space the non-dimensional cell concentrations are associated with a continuum field $u = u(x,t)$. At the edges along the channel width, that is $x=0,1$, zero flux boundary conditions are imposed, corresponding to the inability of cells to pass through these boundaries. Image created with BioRender.com

We study a broad class of problems that are related to the dynamics of a cell culture in microfluidic devices under the influence of a chemoattractant, when the concentration of the agent can be computed or measured. A schematic view of this situation is represented in Fig. 1. The large aspect ratio of the microdevice chamber entails a localised initial seeding along the side labelled *A* in plot (1a) will induce chemotaxis toward side $A'$. This may be represented by a one-dimensional model with axis along the direction $AA'$ representing chemotaxis, with the front representing a continuum model approximation to the edge of the cell population as it migrates towards the high oxygen levels at $A'$. In particular, given a uniform seeding of cells along *A* and high oxygen levels are maintained without variation along the direction of the long side of the microdevice chamber labelled $A'$, then in the direction of this long side there will be no significant gradients of oxygen, and thus no gradients in cell motility and hence cells. Similarly, the small lengthscale of the vertical direction entails an absence of significant gradients vertically. Hence, this geometry with a suitably uniform seeding of initial cell populations and suitably maintained oxygen supply, motivates the consideration of one-dimensional models, with variation in the direction $AA'$, as also motivated by experimental studies (Ayuso et al. 2016, 2017; Ayensa-Jiménez et al. 2020). Hence, we have focussed on one-dimensional models throughout the manuscript.

The cell dynamics in this dimension firstly has an unbiased random motility contribution, as modelled by diffusion, while growth is taken to be logistic, as exemplified by the glioma models of Swanson et al. (2000); Jacobs et al. (2019); Konukoglu et al. (2010). These studies present further detailed model justification, together with model validation in the context of MRI scans tracking tumour evolution. While in central nervous system tissue numerous complexities, ranging from boundaries to the different properties of white and grey matter, entail that a complex process is required to compare simulation and observation (Jacobs et al. 2019), with simple one-dimensional models insufficient, such complications are not present in the microdevice setting. Hence the modelling framework can be considered in the context of the simpler one-dimensional models that we investigate below. However, in the microdevices, there is chemotactic bias in favour of a chemotactic gradient, such as the oxygen profiles in the studies of Ayensa-Jiménez et al. (2020). In controlled microdevice settings the chemotactic concentration may often be assumed as known, for example due to measurement or simulation, with the chemotactic agent often approximately independent of the cell population, for instance due to extensive exogenous supply. Hence, we also consider chemotaxis in the cell motility modelling, represented by the ubiquitous model of Keller and Segal (1971), with a reduction to a known chemoattractant gradient. However, this also entails that cellular migration may not be as simple as a travelling wave, as will be examined below. Finally, while the cell carrying capacity is taken to be fixed, for example by assuming it is imposed due to space limitations, the growth rate may also depend on growth factor or nutrient concentrations and this possibility is also allowed in the modelling framework below.

In summary, the non-dimensional equation for the cell population density, $u\ge 0$, thus represents cellular diffusion together with chemotaxis in response to a known heterogeneous chemoattractant that generates an advective flux $\alpha (t,x)u$, where $\alpha (t,x)$ represents the non-dimensional gradient of the chemotactic agent concentration. Furthermore, cellular proliferation is also heterogeneous, with a constant logistic carrying capacity that has been non-dimensionalised to unity and a heterogeneous growth rate $\beta (t,x)u\ge 0$, where $\beta (t,x)$ represents the modulation of the growth rate by a nutrient, chemoattractant or a growth factor. Thus the non-dimensional governing equations are given by

1 $$\begin{aligned} u_t + \left( \alpha (t,x)u\right) _x = D u_{xx} + \beta (t,x)u(1-u), \end{aligned}$$

with $D>0$ the non-dimensional cellular diffusion coefficient. This governing equation is also supplemented by zero-flux boundary conditions, given by

2a $$\begin{aligned} Du_x-\alpha (t,x)u \big |_{x=0}&= 0, \end{aligned}$$

2b $$\begin{aligned} Du_x-\alpha (t,x)u \big |_{x={1}}&= 0, \end{aligned}$$

which represents the inability of cells to pass through the microdevice chamber walls. Finally, the model is closed by the initial conditions

3 $$\begin{aligned} u(t=0,x)=u_0(x), \end{aligned}$$

where $u_0(x)$ typically represents by a localised seeding of cells from which an invading front emerges.

### Computation of the General Solution for Small Diffusion

#### Outer Solution

The main hypothesis, which is usually true for cellular motility due to weak cell-based random motility, is that the non-dimensional diffusion coefficient satisfies $D \ll 1$, as we verify in Appendix E for the case of GBM cells in microdevices. Hence, away from boundary layers, diffusion may be neglected compared to the influence of growth and chemoattractant driven migration. Then, Eq. (1) may be approximated by:

4 $$\begin{aligned} u_t + (\alpha (t,x)u)_x = \beta (t,x)u(1-u). \end{aligned}$$

**Fig. 2:**
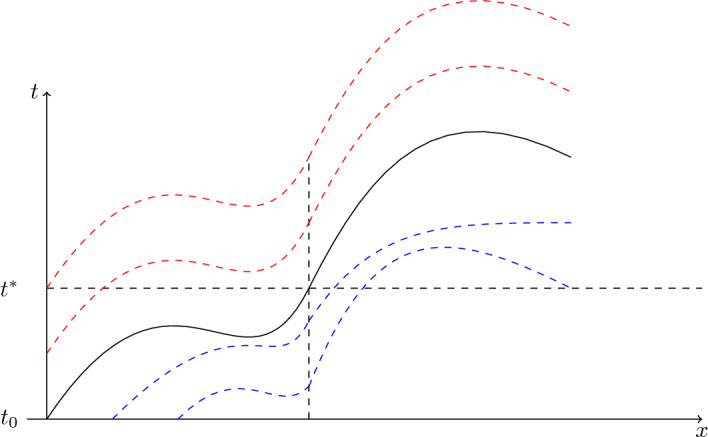
Projection of the characteristic curves. The two families of characteristic curves are shown in blue and red (Color figure online)

This is a first-order hyperbolic PDE, amenable to the method of characteristics. If we know the initial data $u(0,x) = u_0(x)$, we can parameterise the initial data via *s* with the relation $(t,x,u) = (0,s,u_0(s))$. Also, there is another family of characteristic curves, emerging from the (*t*, *x*) points where $x=0$ and $t>0$, with the imposition of the $x=0$ boundary condition of no flux, Eqs. (2). Assuming that $\alpha (t,0) \ne 0$, and that the boundary is away from the transition region of the cellular wavefront, so that to excellent approximation $u_x= 0$ since spatial gradients are small, we conclude from the boundary condition, Eqs. (2), that $u(0,t)=0$ to the same level of approximation. Therefore, this boundary condition can be parameterised via *s* with the relation $(t,x,u) = (s,0,0)$. Hence, at $t=0$ and $x=0$ there is an emerging singular characteristic that splits the domain in two regions. The geometric interpretation of the method of characteristics is shown in Fig. 2, which shows the projection of the characteristic curves onto the plane (*t*, *x*).

We have, therefore, two families of characteristic curves. For the first one:

5a $$\begin{aligned} \frac{\textrm{d}t}{\textrm{d}\tau }&= 1, \quad t(0) = 0, \end{aligned}$$

5b $$\begin{aligned} \frac{\textrm{d}x}{\textrm{d}\tau }&= \alpha (t,x), \quad x(0) = s, \end{aligned}$$

5c $$\begin{aligned} \frac{\textrm{d}u}{\textrm{d}\tau }&= u\left( \beta (t,x)(1-u)-\alpha _x(t,x)\right) , \quad u(0) = u_0(s), \end{aligned}$$

and for the second:

6a $$\begin{aligned} \frac{\textrm{d}t}{\textrm{d}\tau }&= 1, \quad t(0) = s, \end{aligned}$$

6b $$\begin{aligned} \frac{\textrm{d}x}{\textrm{d}\tau }&= \alpha (t,x), \quad x(0) = 0, \end{aligned}$$

6c $$\begin{aligned} \frac{\textrm{d}u}{\textrm{d}\tau }&= u\left( \beta (t,x)(1-u)-\alpha _x(t,x)\right) , \quad u(0) = 0. \end{aligned}$$

Noting the uniqueness of the solution to Eqs. (6) courtesy of Picard’s theorem, we have by inspection that these equations only generate the trivial solution $u(x,t)=0$ for this second set of characteristic curves and thus leading orders predictions of zero cell densities are not uniform but only valid on certain regions of the domain, and away from transitions, as may be seen in Sect. 2.2.3 below for instance.

In contrast, the solution to the first family, given by Eqs. (5), is typically more complex, though one always has

7 $$\begin{aligned} t = \tau . \end{aligned}$$

Progress can be readily made when

- $\alpha (t,x)$ is linear in *x*, such that $\alpha (t,x)=a(t)x+b(t)$.
- $\alpha (t,x)$ is separable, that is $\alpha (t,x)=f(x)g(t)$.

In particular when $\alpha (t,x)$ is linear in *x* we have

8 $$\begin{aligned} x = s \textrm{e}^{\int _0^t a(\eta )\textrm{d} \eta } + \int _0^t b(\eta )\textrm{e}^{\int _{\eta }^t a(\xi )\textrm{d} \xi } \, \textrm{d} \eta =: F(t;s), \end{aligned}$$

which defines *F*(*t*; *s*) for $\alpha (t,x)=a(t)x+b(t)$. We generalise this definition so that $x=F(t;s)$ on the characteristic curve given by the value of *s*, and whenever this relation can be uniquely inverted for *s*, we write $s=G(t;x)$.

In contrast when $\alpha (t,x)=f(x)g(t)$ Eqs. (5a) and (5b) may be integrated to obtain

9 $$\begin{aligned} \int _s^x\frac{\textrm{d}z}{f(z)} = \int _0^\tau g(\eta )\,\textrm{d}\eta . \end{aligned}$$

In turn, Eq. (9) generates the relation $x=F(t;s)$ on the characteristic curve. Further motivation for examples of linear and separable chemotactic response functions for $\alpha (t,x)$ are given in section 3 below.

In both cases, or even in the more general case where *F*(*t*; *s*) cannot readily be determined analytically for all relevant *t*, *s*, the location of the transition from $u=0$, and thus the location of the transition region for the cellular wavefront, $x^* = x^*(t)$, is given by the characteristic with $s=0$ – hence $x^*(t)=F(t;0)$. This is particularly informative about the general behaviour of the solution, for instance in determining the wavespeed. With respect to Eq. (5c), we proceed using the change of variable $r = 1/u$, we obtain the ODE in terms of the $\tau = t$ variable:

10 $$\begin{aligned} r'(\tau )+\left( \beta (\tau ,x(\tau ;s))-h(\tau ;s)\right) r =\beta (\tau ,x(\tau ;s)), \end{aligned}$$

where for the linear case $h(\tau ; s) = a(\tau )$ and $h(\tau ;s) = f'(F(\tau ;s))g(\tau )$ for the separable case.

Noting the integration is along a characteristic, and thus *s* is fixed, this equation is of the form $r'+p(\tau )r=q(\tau )$ for $p(\tau ) =\beta (\tau ,x(\tau ;s))-h(\tau ;s)$ and $q(\tau ) = \beta (\tau ,x(\tau ;s))$, with *s* fixed, so a general expression is given by

11 $$\begin{aligned} r(\tau ,s) = \exp \left( -\int _0^\tau p(\eta ,s) \, \textrm{d}\eta \right) \left[ \frac{1}{u_0(s)} + \int _0^\tau q(\eta ,s) \exp \left( \int _0^\eta p(\xi ,s) \, \textrm{d}\xi \right) \, \textrm{d}\eta \right] ,\nonumber \\ \end{aligned}$$

where $u_0(s)$ is indeed the value of $u_0$ at the location of the characteristic when $\tau =t=0$.

Recapping, suppose $x = F(t;s)$ may be inverted to give $s=G(t;x)$. Then, noting

$$\begin{aligned} x(0,s)=F(0;s)=s, \end{aligned}$$

by the parameterisation of the initial data, we have

$$\begin{aligned} u(x,t) = u(x = F(t;s),t) = \frac{1}{r(t;s = G(t;x))}, \end{aligned}$$

and, in particular

$$\begin{aligned} u_0(s) = u_0(F(0,G(t;x))) = u_0(G(t;x)). \end{aligned}$$

Combining these expressions with Eq. (11), we obtain a general expression for *r* and therefore for $u = u(x,t)$:

12 $$\begin{aligned} u(x,t) = \left( \frac{u_0(s)\exp \left( \int _0^t p(\eta ,s) \, \textrm{d}\eta \right) }{1 + u_0(s)\int _0^t q(\eta ,s) \exp \left( \int _0^\eta p(\xi ,s) \, \textrm{d}\xi \right) \, \textrm{d}\eta }\right) \Bigg |_{s = G(t;x)}, \end{aligned}$$

where $$s=G(t;x) $$ is fixed on each characteristic curve and

$$\begin{aligned} p(\eta ,s)&= \beta (\eta ,F(\eta ;s))-h(\eta ;s), \\ q(\eta ,s)&= \beta (\eta ,F(\eta ;s)). \end{aligned}$$

Eq. (12) may be also written in terms of *x* and *t* directly, obtaining

13 $$\begin{aligned} u(x,t) = \frac{u_0(G(t;x))\exp \left( \int _0^t p(\eta ,G(t;x)) \, \textrm{d}\eta \right) }{1 + u_0(G(t;x))\int _0^t q(\eta ,G(t;x)) \exp \left( \int _0^\eta p(\xi ,G(t;x)) \, \textrm{d}\xi \right) \, \textrm{d}\eta }. \end{aligned}$$

***A special separable case for*** ${\varvec{u(x,t)}}$

Often below, when the chemoattractant flux term is independent of time, so that $\alpha (t,x)=f(x)$, we will have $u_0\ll 1$, $\beta (t,x)\equiv 1$, $g(t)=1$. In these circumstances we have the simplification

$$\begin{aligned} s=G(t;x)=F(-t;x), \end{aligned}$$

by the symmetry $(x,s,\tau )\rightarrow (s,x,-\tau )$ in Eq. (9) with $g(t)=1$ and also that

$$\begin{aligned} p(\eta ,s) = 1 - f'(F(\eta ;s)), \end{aligned}$$

which gives

$$\begin{aligned} u(x,t) = u_0(G(t;x))\textrm{e}^t \exp \left( -\int _0^t f'(F(\eta ,s)) \, \textrm{d}\eta \right) +\mathcal {O}(u_0^2). \end{aligned}$$

Recalling that the integration is along a characteristic, so that *s* is fixed, we change the integration variable via $X= F(\eta ;s)$, noting from Eq. (7) and from Eq. (5b), with *s* fixed and $\alpha (t,x) = f(x)$, that

$$\begin{aligned} \frac{1}{f(X(\eta ))}= \frac{\textrm{d}\eta }{\textrm{d}X}. \end{aligned}$$

Hence, on further noting $s=F(0;s),~x=F(t;s)$ and $s = G(t,x)$, we have, to within $\mathcal {O}(u_0^2)$ corrections, that

14 $$\begin{aligned} u(x,t) = u_0(G(t;x)) \textrm{e}^t \exp \left( -\int _{s}^{x} \frac{f'(X)}{f(X)} \, \textrm{d}X\right) , \end{aligned}$$

so finally

15 $$\begin{aligned} u(x,t)= u_0(G(t;x)) \textrm{e}^t \frac{f(G(t;x))}{f(x)} = u_0(G(t;x)) \textrm{e}^t \frac{f(F(-t;x))}{f(x)}. \end{aligned}$$

In the examples plotted below we also have $$u_0(x)=u_0$$ is constant, in which case $$ u_0(G(t;x))$$ collapses to the constant $$u_0$$. For clarity, please note that $$u_0$$ is an abbreviation for $$ u_0(G(t;x))$$ in general, though this is constant and denoted simply by $$u_0$$ in all examples, separable or otherwise, plotted below.

#### Inner Solution

To explore the transition layer moving with the wavefront, we introduce a scaling of coordinates such that diffusion and advection provide a leading order dominant balance in the transition layer, with $x=x^*(t)+\delta X$ for *X* the inner variable and $\delta \ll 1$. With the change of variables

$$\begin{aligned} (t,x)\rightarrow (\tau , X), \quad t=\delta \tau , \quad x=x^*(t)+\delta X, \quad U( \tau ,X)=u(t,x), \end{aligned}$$

one has the inner solution equations

$$\begin{aligned} \frac{1}{\delta }U_\tau= & {} \frac{1}{\delta }\left( x_t^*-\alpha (t,x^*(t)+\delta X ) \right) U_X + \frac{D}{\delta ^2}u_{XX} +\beta U(1-U) - \alpha _x(t,x^*(t)+\delta X) \\ {}= & {} \frac{1}{\delta }\left( \alpha (t,x^*(t) ) -\alpha (t,x^*(t)+\delta X ) \right) U_X + \frac{D}{\delta ^2}U_{XX} +\mathcal {O}(1), \end{aligned}$$

where the second line uses $x_t^*=\alpha (t,x^*(t) )$. We take $\delta =D \ll 1$ to bring the advective and diffusive terms into a nominal dominant balance.

Let us now assume that $\alpha$ uniformly possesses an order one derivative with respect to *x*, that is, we assume that $\alpha (t,x^*(t)) - \alpha (t,x^*(t) + \delta X) \simeq -\alpha _x(t,x^*(t))\delta X$, therefore

$$\begin{aligned} U_{\tau } = u_{XX} +\mathcal {O}(\delta ), \end{aligned}$$

for $X\sim \mathcal {O}(1)$. The solution of this equation, when $U(X,\tau =0) = H(X)$, with *H* the Heaviside step function, is

$$\begin{aligned} U(X,\tau ) = \frac{1}{2}\left( 1+\textrm{erf}\left( \frac{X}{2\sqrt{\tau }}\right) \right) . \end{aligned}$$

#### Composite Solution

We can combine the inner solution, rewritten in terms of (*x*, *t*) with the outer solutions via a composite approximation to generate an approximation for the full numerical solution across the domain (away from any prospective boundary layer at $x=1$). Thus, with $u_0(x)=u_0$, constant, and $u_{\textrm{an}}(x,t)$ the analytical characteristic solution of Eq. (13) we have

16 $$\begin{aligned} u(x,t) \sim \left\{ \begin{array}{lll} u_{\textrm{an}}(x^*(t),t)U\left( \frac{x-x^*(t)}{D},\frac{t}{\tau }\right) + &{}0, &{} \quad x< x^*(t), \\ u_{\textrm{an}}(x^*(t),t)U\left( \frac{x-x^*(t)}{D},\frac{t}{\tau }\right) + &{}\left( u_\textrm{an}(x,t) - u_\textrm{an}(x^*(t),t)\right) , &{} \quad x\ge x^*(t), \end{array} \right. \nonumber \\ \end{aligned}$$

so, finally

17 $$\begin{aligned} u(x,t) \sim \left\{ \begin{array}{ll} \frac{1}{2}u_{\textrm{an}}(x^*(t),t)\left( \textrm{erf}\left( \frac{x-x^*(t)}{2\sqrt{tD}}\right) +1\right) , &{} \quad x< x^*(t), \\ \frac{1}{2}u_{\textrm{an}}(x^*(t),t)\left( \textrm{erf}\left( \frac{x-x^*(t)}{2\sqrt{tD}}\right) -1\right) + u_\textrm{an}(x,t), &{} \quad x\ge x^*(t). \end{array} \right. \nonumber \\ \end{aligned}$$

For the special case discussed in the previous section, that is, when $\alpha (t,x) = f(x)$, $\beta (t,x) \equiv 1$ and $u_0 \ll 1$, $u_\textrm{an}(x,t) = u_0(G(t;x))\textrm{e}^t\frac{f(G(t;x))}{f(x)}$ and in particular $u_\textrm{an}(x^*(t),t) = u_0(0)\textrm{e}^t\frac{f(0)}{f(x)}$ so that for this special case

18 $$\begin{aligned} u(x,t) \sim \left\{ \begin{array}{ll} \frac{1}{2}u_0(0)\textrm{e}^t\frac{f(0)}{f(x)}\left( \textrm{erf}\left( \frac{x-x^*(t)}{2\sqrt{tD}}\right) +1\right) , &{} \quad x< x^*(t), \\ \frac{1}{2}u_0(0)\textrm{e}^t\frac{f(0)}{f(x)}\left( \textrm{erf}\left( \frac{x-x^*(t)}{2\sqrt{tD}}\right) -1\right) + u_0(G(t;x))\textrm{e}^t\frac{f(G(t;x))}{f(x)}, &{} \quad x\ge x^*(t), \end{array} \right. \nonumber \\ \end{aligned}$$

with continuity assured from the constraint $G(t;x^*(t))=0$, which holds as, by construction, both $s=0$ and $x=x^*(t)$ hold on the separating characteristic and $s=G(t;x)$.

We note that this analytical solution will lose accuracy, typically severely so, within the localised vicinity of the right-hand boundary, that is $x=1$. In particular, we have an additional boundary condition at this location, where the right-hand outer solution is imposed for the solutions of Eqs. (17), (18) above, with $x\ge x_*(t)$. This outer solution, emerging from a hyperbolic equation with $D=0$, does not have enough degrees of freedom to satisfy the additional boundary condition and hence the breakdown in accuracy. For the $x=1$ boundary condition to be satisfied, sufficiently large gradients in cell density are required with diffusive motility becoming a leading order effect which provides the necessary freedom. This a classical boundary layer, as documented in numerous textbooks, such as Bender and Orszag (2013), with this specific example of a boundary layer studied extensively in Morton’s (1996) textbook. An analogous boundary exists close to $x=0$ for sufficiently small time, much smaller than the non-zero time results presented. As with all boundary layers, the boundary layers are localised and do not impact the rest of the solution and thus we do not consider them further, except to remark that accuracy is lost sufficiently close to $x=1$ as appropriate in the presentation of the results below. Analogous statements apply sufficiently near $x=0$ for sufficiently small time, but such times are so small that this $x=0$ boundary layer is not captured in the presented results, which are unaffected by this short-time dynamics.

### Particular Cases of Interest

The solution given by Eq. (13) is general given that $D \ll 1$, though even the separable case using Eq. (9) to determine the functions *F* and *G* can generate complicated solutions that are not readily expressed in terms of standard functions. Further, even when $\alpha (t,x)$ is separable or linear, allowing extensive analytical progress, there is still considerable freedom in the form of $\alpha (t,x)$ and hence we firstly analyse exemplar case of chemotaxis in the presence of heterogeneity, before proceeding to consider an example of cellular behaviour in a microdevice.

We consider the case of heterogeneous chemotaxis induced by the chemoattractant spatial distribution. In particular, we derive a solution for linear gradients and for exponential gradients that are modulated in time, with exponential gradients emerging when there are point sources. A third case, considering quadratic gradients, is also presented in Appendix A for reference.

The accuracy of the different particular solutions derived is evaluated by comparing them to results from the Matlab pdepe routine, which numerically approximates the solution of initial-boundary value problems for systems of parabolic and elliptic partial differential equations in one space variable and time, noting here the full model is parabolic since $D>0$. This Matlab routine uses a time-space integrator based on a piece-wise nonlinear Galerkin approach which is second-order accurate in space (Skeel and Berzins 1990). For a quantitative comparison between the numerical and analytical results obtained, the reader is referred to Appendix c.

#### Linear Gradients

We first consider a function of the form

$$\begin{aligned} \alpha (t,x) = (ax+b)g(t). \end{aligned}$$

Then, we have $f(x)=ax+b$ and Eq. (9) yields

$$\begin{aligned} \frac{1}{a} \ln \left( \frac{ax+b}{as+b}\right) = \int _0^\tau g(\eta )\,\textrm{d}\eta =: {{\mathcal {T}}}(\tau ). \end{aligned}$$

Thus defines the function ${{\mathcal {T}}}$, and with ${{\mathcal {T}}}(t) = \int _0^t g(\eta )\,\textrm{d}\eta$ we have

$$\begin{aligned} F(t;s)&= \frac{1}{a}\left[ (as+b)\textrm{e}^{a {{\mathcal {T}}}(t) }-b\right] , \\ G(t;x)&= \frac{1}{a}\left[ (ax+b)\textrm{e}^{-a {{\mathcal {T}}}(t) }-b\right] . \end{aligned}$$

Hence, the transition is located at

$$\begin{aligned} x^*(t) = \frac{b}{a}\left( \textrm{e}^{a {{\mathcal {T}}}(t) }- 1\right) . \end{aligned}$$

**Fig. 3:**
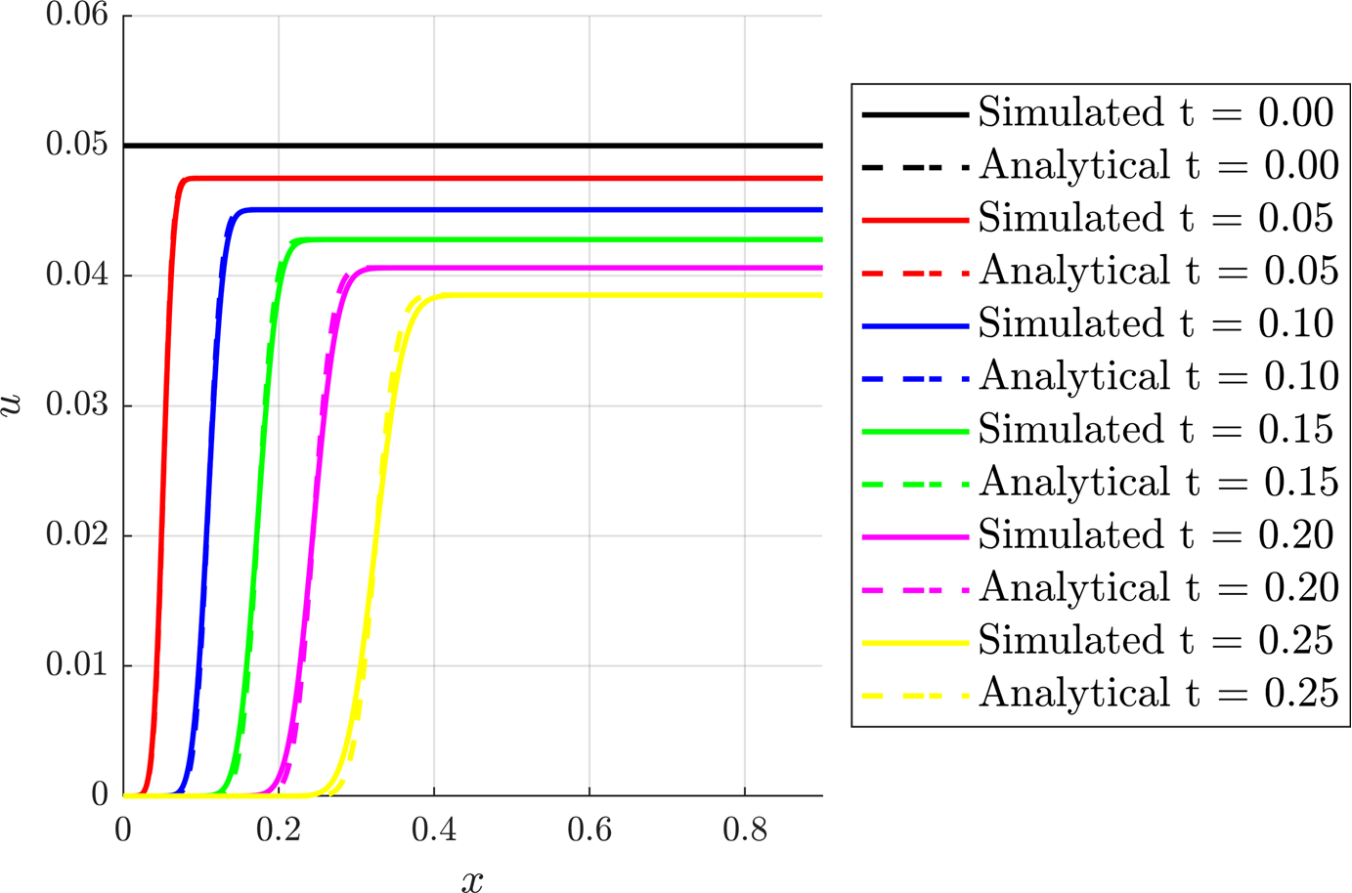
(Color Figure Online) Comparison of numerical and analytical solutions for the linear case. The analytical and simulated profiles are compared at different times, considering $a = 2$ and $b = 1$, $\beta (t,x) = g(t) = 1$ and $u_0(x) = u_0 = 0.05$, whereas the full numerical simulation was obtained using $D=1\times 10^{-3}$. For this plot, and for analogous plots below, there are localised boundary layer effects near the right-hand edge of the domain, $x=1$, that are not captured by the presented analytical solution. An analogous boundary layer is present in the immediate vicinity of $x=0$ for sufficiently small time, such that the chemotactic wavefront has not evolved to fully move away from the $x=0$ boundary, and thus a significantly smaller time than the non-zero times presented here. Further details concerning boundary layers have been described in the final paragraph of Sect. 2.2.3

Furthermore, for $\beta (t,x) = 1$ and $g(t)=1$, so that $\mathcal{T}(t)=t$, the integral expression for *u*(*x*, *t*) in Eq. (13) is readily determined to reveal

$$\begin{aligned} u(x,t) = \frac{ u_0 (a-1) \textrm{e}^{-(a-1)t}}{a-1+u_0 \left( 1- \textrm{e}^{-(a-1)t} \right) } = u_0 \textrm{e}^{-(a-1)t} +\mathcal {O}(u_0^2), \end{aligned}$$

while $u=0$ before the transition. Figure 3 shows a comparison between the numerical results with $D=1\times 10^{-3}$ and the approximate analytical solution for $\alpha (x)=2x+1$ and $\beta (t,x) = 1 = g(t)$. For this plot, and for analogous plots below, note the accurate prediction of the evolving front, $x^*(t)$ and the general agreement between the numerical and analytical solutions for *u*(*x*, *t*). Furthermore, one can expect boundary layer effects near the right-hand edge of the domain, $x=1$, that are not captured by the presented solution, though these are not plotted in the current figure.

A further, and particularly relevant case, is when $g(t)=\cos (\omega t)$, whence

$$\begin{aligned} F(t;s)&= \frac{(as+b)\exp \left( \frac{a}{\omega }\sin (\omega t)\right) -b}{a}, \\ G(t;x)&= \frac{(ax+b)\exp \left( -\frac{a}{\omega }\sin (\omega t)\right) -b}{a}. \end{aligned}$$

Note we still have $G(t;x) = F(-t,x)$ since $g(t)=\cos (\omega t)$ is even so that $(x,s,\tau )\rightarrow (s,x,-\tau )$ remains a symmetry of Eq. (9). The transition is located at

$$\begin{aligned} x^*(t) = \frac{b}{a}\left[ \exp \left( \frac{a}{\omega }\sin (\omega t)\right) -1\right] . \end{aligned}$$

**Fig. 4:**
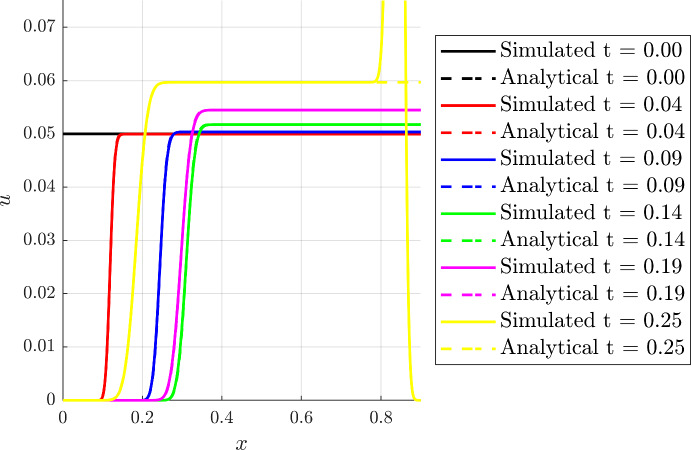
(Color Figure Online) Comparison of numerical and analytical solutions for the linear case with oscillatory stimulus. The analytical and simulated profiles at different times are compared, considering $a=1$, $b=3$, $\omega = 10$, $\beta (t,x) = 1$ and $u_0(x) = u_0 = 0.05$ whereas the full numerical simulation was again obtained using $D=1\times 10^{-3}$. One can clearly observe that the wave of cells oscillates, with the cell density spatially constant on the right of the transition, except on approaching $x=1$, where there is a boundary layer that is not accommodated in the analysis. An analogous boundary layer is present in the immediate vicinity of $x=0$ for sufficiently small time, that is significantly smaller than the non-zero times presented here. Further details concerning boundary layers have been described in the final paragraph of Sect. 2.2.3

Figure 4 shows the comparison between the numerical results with $D=1\times 10^{-3}$ and the analytical solution, with $\beta (t,x) = 1$. Note that as almost all of the domain is displayed, the boundary layer in the numerical solution near $x=1$ can be observed.

The oscillating gradient case is very pertinent as it corresponds to the case where the oxygenation feed between the two channels at the microfluidic device switches, so we shall explore it in more detail. Rather than working with the general solution given by Eq. (13) it can be more expedient to consider the differential equation for $r=1/u$ given by Eq. (10); noting $\beta =1$, $f(x)=(ax+b)$, $u_0(x)=u_0$, constant, for the cases considered here, this reduces to

19 $$\begin{aligned} r'+\left( 1-a\cos (\omega t)\right) r = 1, \quad r(0) = 1/u_0, \end{aligned}$$

with no *x*-dependence. Hence, on the right of the transition region the solution is constant, that is, $u(x) = u^*$.

In Appendix 1 we show that Eq. (19) may be solved using different asymptotic methods for four different regimes:

- Slow variations of the gradients, $\omega \ll 1$.
- Fast variations of the gradients, $\omega \gg 1$.
- Dominant chemotaxis, $$\beta \ll a$$.
- Dominant growth, $$a \ll \beta $$.

**Fig. 5:**
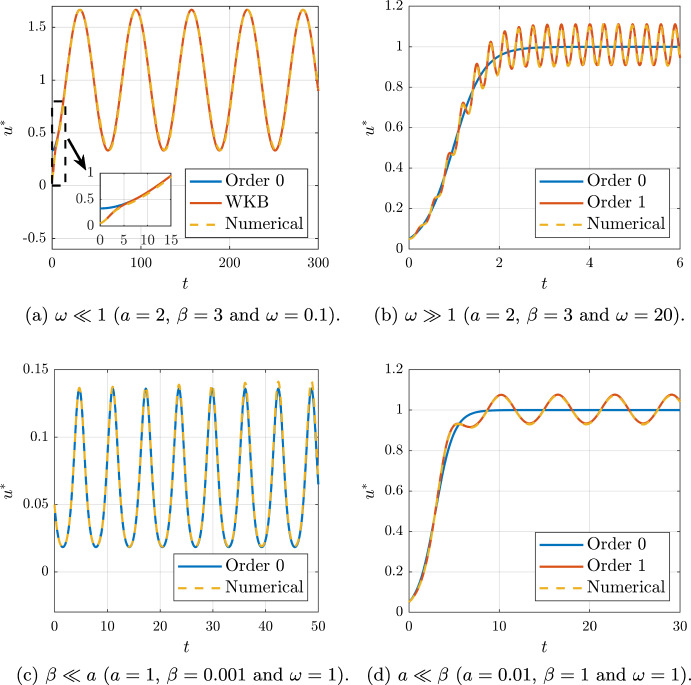
(Color Figure Online) Comparison between analytical solutions using asymptotic theory and numerical solutions for the oscillatory gradient. The four exposed cases are analysed: slow variations of the gradients ($\omega \ll 1$), fast variations of the gradients ($\omega \gg 1$), dominant chemotaxis ($\beta \ll a$) and dominant growth ($a \ll \beta$)

For spatial locations to the right of the transition region, but away from any boundary layer at $x=1$, these approximate, time-oscillating, solutions are compared to numerical solutions computed using standard Runge–Kutta solvers for Eqn. (19) in Fig. 5.

#### Exponential Gradients

We consider now a function of the type:

$$\begin{aligned} \alpha (t,x) = \left( a\exp (-\lambda x)+b\exp (\lambda x)\right) g(t). \end{aligned}$$

Then, we have $f(x)=a\exp (-\lambda x)+b\exp (\lambda x)$, with Eq. (9) reducing to

$$\begin{aligned} \arctan \left( \frac{b\exp (\lambda x)}{\sqrt{ab}}\right) - \arctan \left( \frac{b\exp (\lambda s)}{\sqrt{ab}}\right) = \sqrt{ab}\lambda \int _0^\tau g(\eta )\,\textrm{d}\eta = \sqrt{ab}{\mathcal {T}}(\tau ). \end{aligned}$$

Hence

$$\begin{aligned} F(t;s)&= \frac{1}{\lambda }\ln \left( \frac{\sqrt{ab} + a\tan \left( \sqrt{ab}\lambda {{\mathcal {T}}}(t) \right) \textrm{e}^{-\lambda s}}{\sqrt{ab}\textrm{e}^{-\lambda s} -b\tan \left( \sqrt{ab}\lambda {{\mathcal {T}}}(t) \right) }\right) , \\ G(t;x)&= \frac{1}{\lambda }\ln \left( \frac{\sqrt{ab} - a\tan \left( \sqrt{ab}\lambda {{\mathcal {T}}}(t) \right) \textrm{e}^{-\lambda x}}{\sqrt{ab}\textrm{e}^{-\lambda x} + b\tan \left( \sqrt{ab}\lambda {{\mathcal {T}}}(t) \right) }\right) . \end{aligned}$$

with the transition is located at

$$\begin{aligned} x^*(t) = \frac{1}{\lambda }\ln \left( \frac{\sqrt{ab} + a\tan \left( \sqrt{ab}\lambda {{\mathcal {T}}}(t) \right) }{\sqrt{ab} -b\tan \left( \sqrt{ab}\lambda {{\mathcal {T}}}(t) \right) }\right) . \end{aligned}$$

In particular, if $b = 0$ this reduces to

$$\begin{aligned} F(t;s)&= \frac{1}{\lambda }\ln \left( a\lambda {{\mathcal {T}}}(t) + \textrm{e}^{\lambda s}\right) , \\ G(t;x)&= \frac{1}{\lambda }\ln \left( -a\lambda {{\mathcal {T}}}(t) + \textrm{e}^{\lambda x}\right) , \end{aligned}$$

and

$$\begin{aligned} x^*(t) = \frac{1}{\lambda }\ln \left( 1+\lambda a {{\mathcal {T}}}(t) \right) . \end{aligned}$$

**Fig. 6:**
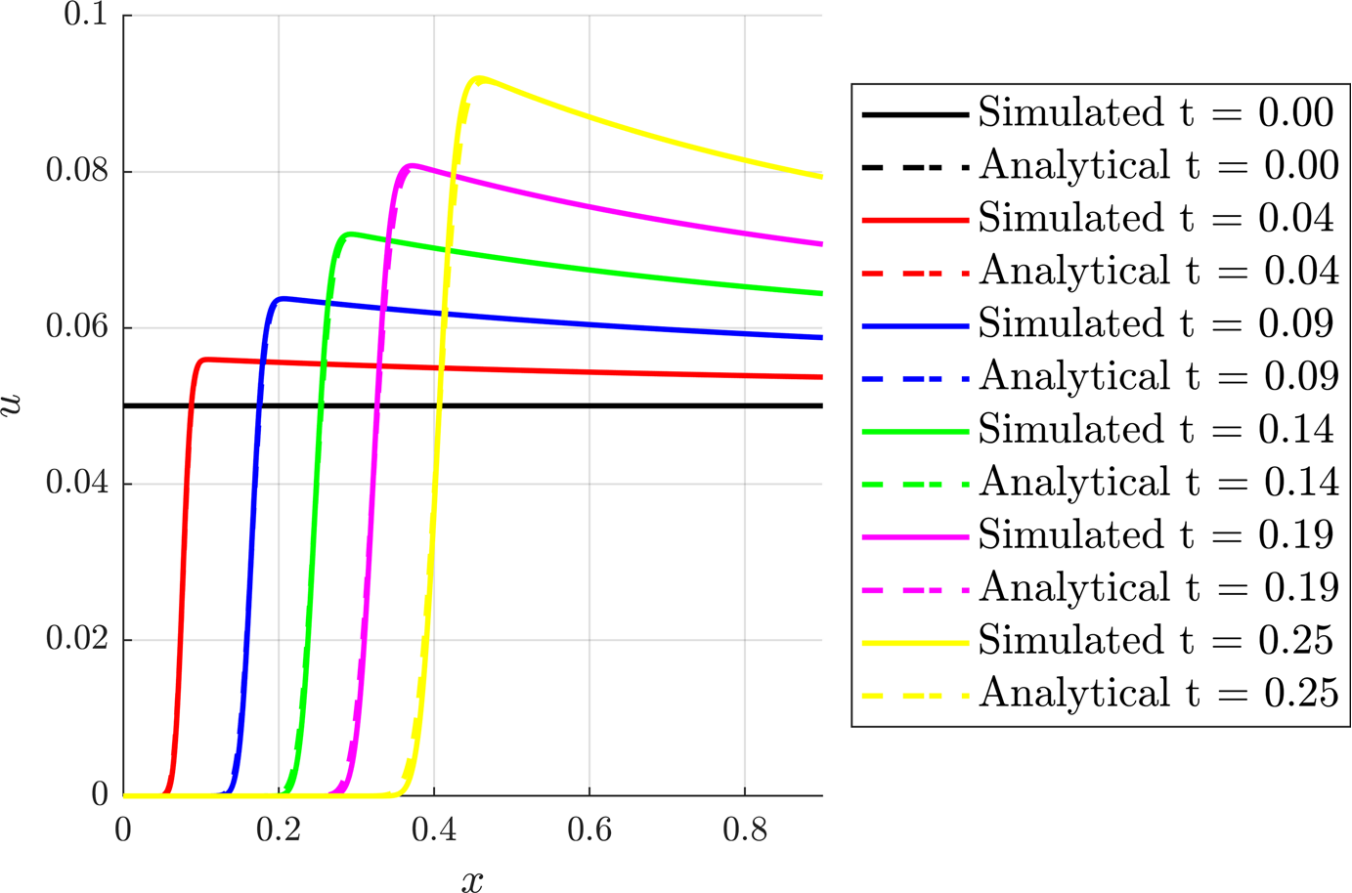
(Color Figure Online) Comparison of numerical and analytical solutions for the exponential case. The analytical and simulated profiles are compared at different times, considering $\beta (t,x)=1=g(t)$, $\alpha (t,x) = a\exp (-\lambda x)$ with $a = 2$, $\lambda = 1$ and the initial condition $u_0(x)=u_0=0.05 \ll 1$. In that case, we use $D = 1 \times 10^{-3}$ for computing the numerical solutions. There are also localised boundary layer effects near the right-hand edge of the domain, $x=1$, that are not captured by the presented analytical solution, with an analogous boundary layer is present in the immediate vicinity of $x=0$ for sufficiently small time, that is significantly smaller than the non-zero times presented here. Further details concerning boundary layers have been described in the final paragraph of Sect. 2.2.3

Figure 6 shows the comparison between the numerical results and the analytical solution with $\alpha (t,x) = 2\exp (-x)$ and $\beta (t,x) = 1=g(t)$, $D=1\times 10^{-3}$. Furthermore, in this regime on neglecting $\mathcal {O}(u_0^2)$ corrections, we have

$$\begin{aligned} u(x,t) = u_0\textrm{e}^t \frac{ f(s)}{f(x)} \bigg |_{s=G(t;x)} = \frac{u_0 \textrm{e}^t}{1-a\lambda t \textrm{e}^{-\lambda x}}, \end{aligned}$$

noting that $x\ge x_*(t)$ on this characteristic, so that

$$\begin{aligned} 1-a \lambda t \textrm{e}^{-\lambda x} \ge 1- a \lambda t \textrm{e}^{-\lambda x_*(t)} = 1- \frac{a\lambda t}{1+a\lambda t}= \frac{1}{1+a\lambda t} >0, \end{aligned}$$

in turn demonstrating that *u*(*x*, *t*) does not possess a singularity with exponential decay in *x* for *t* fixed away from the transition to a good approximation, as may be also observed in Fig. 6.

## Applications to Potential Microfluidic Experiments

### The General Model

We study a broad class of problems that are related to the evolution of a cell culture in microfluidic devices under a chemotactic agent, such as oxygen, when the concentration of the agent can be computed or measured, as schematically represented in Fig. 1. Hence, we proceed to consider the following model of generic cell culture evolution in microfluidic devices (Ayensa-Jiménez et al. 2020; Ayensa-Jiménez 2022)

20a $$\begin{aligned} \frac{\partial C_n}{\partial T}&= \frac{\partial }{\partial X}\left( D\frac{\partial C_n}{\partial X} - \chi C_n\frac{\partial B}{\partial X}\right) + \alpha _n M_g(B)C_n\left( 1-\frac{C_n}{c_\textrm{sat}}\right) - \alpha _{nd}M_d(B)C_n, \end{aligned}$$

20b $$\begin{aligned} \frac{\partial C_d}{\partial T}&= \alpha _{nd}M_d(B)C_n, \end{aligned}$$

20c $$\begin{aligned} \frac{\partial B}{\partial T}&= \frac{\partial }{\partial X}\left( D_B\frac{\partial B}{\partial X}\right) - \alpha _B W(B,C_n). \end{aligned}$$

where $C_n$ and $C_d$ are respectively the alive and dead cell concentrations and *B* is a chemotactic agent, $M_g$ and $M_d$ are nonlinear dimensionless corrections accounting for the effect of the chemo-attractant on cell growth and death. In addition $W(B,C_n)$ is the nonlinear dimensionless correction of the chemo-attractant consumption by all cells in the microdevice, noting that additional cells other than those of direct interest may be present, such as the study of metastatic tumour cells of interest migrating within a stromal cell population for instance. We also assume that the dead cell concentration is sufficiently low to ensure that it does not compromise either live cell proliferation or migration. Note that if the chemotaxis agent is a cell nutrient, such as glucose or oxygen, $W(B,C_n) \ne 0$, whereas for unconsumed biochemical signals, $W(B,C_n)=0$. The boundary conditions considered are

21a $$\begin{aligned} {f_n(x=0,t)}&= 0, \end{aligned}$$

21b $$\begin{aligned} {f_n(x=L,t)}&= 0, \end{aligned}$$

21c $$\begin{aligned} B(x=0,t)&= B^L(t), \end{aligned}$$

21d $$\begin{aligned} B(x=L,t)&= B^R(t), \end{aligned}$$

with $B^L(t)$ and $B^R(t)$, the chemotactic agent concentration at the left and right channel, and $f_n = D\frac{\partial C_n}{\partial X} - \chi C_n\frac{\partial B}{\partial X}$ the live cell flux. As the dead cell population is slave to the other variables in this model, we neglect it henceforth which is equivalent to taking $\alpha _{nd} \simeq 0$.

Therefore, the full model here analysed is

22a $$\begin{aligned} \frac{\partial C_n}{\partial T}&= \frac{\partial }{\partial X}\left( D\frac{\partial C_n}{\partial X} - \chi C_n\frac{\partial B}{\partial X}\right) + \alpha _n M_g(B) C_n \left( 1-\frac{C_n}{c_{\textrm{sat}}}\right) , \end{aligned}$$

22b $$\begin{aligned} \frac{\partial B}{\partial T}&= \frac{\partial }{\partial X}\left( D_B\frac{\partial B}{\partial X}\right) - \alpha _B W(B,C_n). \end{aligned}$$

In order to evaluate the relevance of the different phenomena, we define the dimensionless variables:

23a $$\begin{aligned} C_n&= c_\textrm{sat}u, \end{aligned}$$

23b $$\begin{aligned} B&= B^*v, \end{aligned}$$

23c $$\begin{aligned} X&= Lx, \end{aligned}$$

23d $$\begin{aligned} T&= \frac{t}{\alpha _n}. \end{aligned}$$

Hence Eqs. (22) become

24a $$\begin{aligned} \frac{\partial u}{\partial t}&= \frac{\partial }{\partial x}\left( \Pi _1\frac{\partial u}{\partial x} - \Pi _2 u\frac{\partial v}{\partial x}\right) + m(v)u\left( 1-u\right) , \end{aligned}$$

24b $$\begin{aligned} \frac{\partial v}{\partial t}&= \frac{\partial }{\partial x}\left( \Pi _3\frac{\partial v}{\partial x}\right) + \Pi _4 w(v,u), \end{aligned}$$

where

25a $$\begin{aligned} \Pi _1&= \frac{D}{\alpha _n L^2}, \end{aligned}$$

25b $$\begin{aligned} \Pi _2&= \frac{\chi B^*}{\alpha _n L^2}, \end{aligned}$$

25c $$\begin{aligned} \Pi _3&= \frac{D_B}{\alpha _n L^2}, \end{aligned}$$

25d $$\begin{aligned} \Pi _4&= \frac{\alpha _B c_{\textrm{sat}}}{ \alpha _n B^*}, \end{aligned}$$

25e $$\begin{aligned} m(v)&= M_g(B^*v), \end{aligned}$$

25f $$\begin{aligned} w(v,u)&= W(B^*v,c_{\textrm{sat}}u). \end{aligned}$$

The associated boundary conditions are

26a $$\begin{aligned} {f_n(x=0,t)}&= 0, \end{aligned}$$

26b $$\begin{aligned} {f_n(x=1,t)}&= 0, \end{aligned}$$

26c $$\begin{aligned} v(x=0,t)&= \psi _1(t), \end{aligned}$$

26d $$\begin{aligned} v(x=1,t)&= \psi _2(t), \end{aligned}$$

where now $f_n = \Pi _1\frac{\partial u}{\partial x} - \Pi _2 u\frac{\partial v}{\partial x}$ and $\psi _1(t),~\psi _2(t)$ are prescribed functions of time corresponding to the level of nutrient or chemoattractant fixed to be at the channel edges. In what follows, we will use $u_t$ and $u_x$ as an abbreviation for $\frac{\partial u}{\partial t}$ and $\frac{\partial u}{\partial x}$ and similarly for $u_{xx}.$

In particular the governing PDEs described in Eqs. (24) may be reformulated as

27a $$\begin{aligned} u_t&= \Pi _1 u_{xx} -\Pi _2 \left( v_x u\right) _x + m(v)u\left( 1-u\right) , \end{aligned}$$

27b $$\begin{aligned} v_t&= \Pi _3 v_{xx} - \Pi _4 w(v,u), \end{aligned}$$

and typical parameter values are considered in Appendix E, where it is noted that $\Pi _1\ll 1$ and $\Pi _3\gg 1$ for instance.

#### The Weak Consumption Limit

First, we consider the case where the chemoattractant is not a nutrient and therefore is not consumed by cells. In that case, we can set

$$\begin{aligned} w(v,u) = 0. \end{aligned}$$

For all the problems in which it is possible to assume $\Pi _1 \ll 1$, whereby random cellular motility is negligible compared to directed chemotaxis and with $\Pi _3 \gg 1$, so that the chemoattractant diffusion is large relative to cellular diffusion Eqs. (27) reduce to

28a $$\begin{aligned} u_t + k \left( v_x u\right) _x&= m(v)u\left( 1-u\right) , \end{aligned}$$

28b $$\begin{aligned} v_{xx}&= 0. \end{aligned}$$

where $$k = \Pi _2$$.

However, care is required in considering the boundary conditions for *u* and the initial conditions for *v* in this reduced model, due to the loss of the second spatial derivative of *u* and the first temporal derivative of *v*. In particular, we cannot satisfy all the boundary conditions for *u*; instead we have boundary layers. We have seen in the examples these occur at internal transitions and at the right of the domain (see Fig. 4). Thus for the simplified system the boundary condition is enforced at $x=0$ for *u* with the simplification of the flux to $f_n=-kv_x u$, as diffusion is treated as negligible. However, enforcing the boundary condition at $x=1$ will require the consideration of a boundary layer that is not resolved in the simplified model as it is complicated, but does not further insight into cell migratory and chemotaxis. The two boundary conditions for *v*, as given by Eqs. (26c) and (26d) are inherited and applied at both boundaries. We now consider the initial conditions for *v*, which cannot be satisfied. Instead there is an analogous temporal boundary layer for early time while initial transients relax, though such transients persist for such a short time that they are not of interest, and thus not resolved, here. The justification of the neglect of these fast transients is further detailed in Appendix D, where it is demonstrated that the solution of Eq. (28b) corresponds to the leading order composite solution in a temporal boundary layer analysis that exploits $\Pi _3\gg 1$.

Proceeding, we set $v(x=0,t) = \psi _1(t)$ and $v(x=1,t) = \psi _2(t)$, Eq. (28b) is immediately integrated to

29 $$\begin{aligned} v(t,x)=\left( \psi _2(t) - \psi _1(t)\right) x + \psi _1(t). \end{aligned}$$

We recover therefore Eq. (4) with

$$\begin{aligned} \alpha (t,x) = k(\psi _2(t) - \psi _1(t)),\quad \beta (t,x) = m(\left( \psi _2(t) - \psi _1(t)\right) x + \psi _1(t)), \end{aligned}$$

that is a special case of the linear problem $\alpha (t,x) = a(t)x+b(t)$, with $a(t) = 0$ and $b(t) = k(\psi _2(t) - \psi _1(t))$, so the different functions needed in order to compute the solution are:

30a $$\begin{aligned} F(t;s)&= s + k\int _0^t\Delta \psi (\eta )\, \textrm{d}\eta , \end{aligned}$$

30b $$\begin{aligned} G(t;x)&= x - k\int _0^t\Delta \psi (\eta )\, \textrm{d}\eta , \end{aligned}$$

30c $$\begin{aligned} x^*(t)&= k\int _0^t\Delta \psi (\eta )\, \textrm{d}\eta = x-G(t;x), \end{aligned}$$

where we have defined $\Delta \psi (t) = \psi _2(t) - \psi _1(t)$. Also, the expression of the cell profile far from the transition is

31 $$\begin{aligned} u(x,t) = \frac{u_0( G(t;x))\exp \left( \int _0^t K(\eta , F(\eta ;G(t;x))) \, \textrm{d}\eta \right) }{1+u_0( G(t;x))\left( \exp \left( \int _0^t K(\eta , F(\eta ;G(t;x)))\, \textrm{d}\eta \right) - 1\right) }, \end{aligned}$$

where

$$\begin{aligned} K(\eta ,X) = m\left( (\psi _2(\eta ) - \psi _1(\eta ))X + \psi _1(\eta )\right) . \end{aligned}$$

**Fig. 7:**
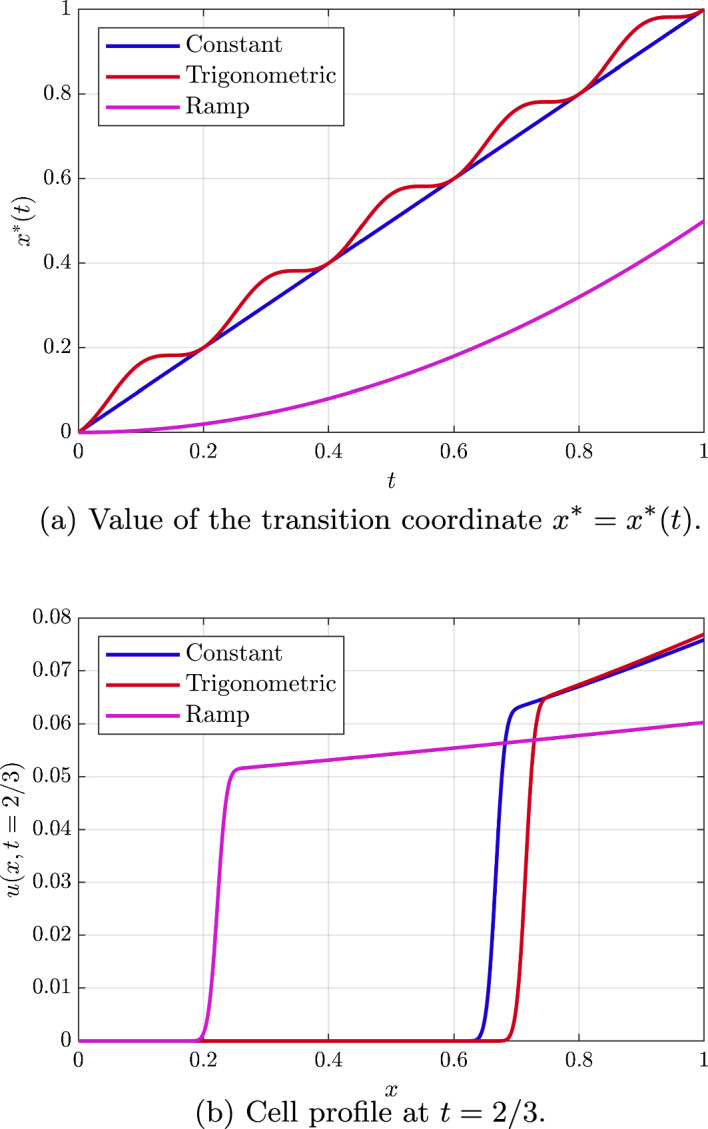
(Color Figure Online) Approximate analytical solution for the case with no consumption. We assume $k=1$, $m(v) = v$ and the transition region is recreated with $D = 1 \times 10^{-4}$. The initial condition is set again to $u_0(x) = u_0 = 0.05$. Also, we consider three different shapes for $\psi _2$, with $\psi _1=0$. A constant oxygen level $\psi _2(t) = 1$, a trigonometric oscillatory stimulus $\psi _2(t) = 1 + \cos (\omega t)$ with $\omega = 10\pi$, and an increasing ramp stimulus $\psi _2(t) = t$

The evolution of the transition coordinate $x^*(t)$ and the dimensionless cell profile for different times are shown in Fig. 7 for $k=1$, $\psi _1(t) = 0$ and different external stimuli $\psi _2(t)$. In particular, let us consider the case with $m(v) = m_0 v$, and with $\psi _1(t)=0$ and use of the change of variable $X=F(\eta ;s)$, whereby on a characteristic

$$\begin{aligned} \dfrac{\textrm{d}X}{\textrm{d}\eta } = \alpha (\eta ,X)=k\psi _2(\eta ) \end{aligned}$$

with $F(t;s)=x$ and $F(0,s)=s$. This reveals

$$\begin{aligned} \int _0^t K(\eta ,F(\eta ,s))\, \textrm{d}\eta \big |_{s=G(t;x)}= & {} \int _s^x \frac{1}{k\psi _2(\eta )} m_0 \psi _2(\eta ) X \, \textrm{d} X \big |_{s=G(t;x)} \\ = \frac{m_0}{2k} (x-G(t;x))(x+G(t;x))= & {} \frac{m_0}{2k} x^*(t)(2x-x^*(t)). \end{aligned}$$

Combined with Eq. (31), this gives a simple expression for *u*(*x*, *t*). For instance

$$\begin{aligned} u(x,t) \approx u_0 \exp \left( \frac{m_0}{k} x^*(t)(x-x^*(t)/2) \right) +\mathcal {O}(u_0^2), \end{aligned}$$

for $u_0\ll 1$ constant; in this case, we have an increasing function at fixed *t* to the right of the transition given $x^*(t)>0$ and this is essentially linear for $x^*(t)\ll 1$, as observed in Fig. 7.

#### Cellular Consumption of Chemoattractant

A more interesting case is when the chemoattractant is a nutrient and therefore it is consumed by cells. In that case, $w(v,u) \ne 0$.

With $w_{\textrm{pc}}(v)$ denoting the non-dimensional uptake of nutrient *per cell*, we take $w_{\textrm{pc}}(v)$ to be monotonic increasing with

$$\begin{aligned} \lim _{v\rightarrow 0} w_{\textrm{pc}}(v) = 0, \\ \lim _{v\rightarrow +\infty } w_{\textrm{pc}}(v) = 1, \end{aligned}$$

where the final limit is without loss of generality, with the overall scale of uptake governed by $\Pi _4$.

For instance, with Michaelis-Menten kinetics we take (Cornish-Bowden 2013):

32 $$\begin{aligned} w_{\textrm{pc}}(v) = \frac{v}{v+k_m}, \end{aligned}$$

or, more generally, we can use the Hill-Langmuir equation for modelling the consumption kinetics (Hill 1910)

33 $$\begin{aligned} w_{\textrm{pc}}(v) = \frac{v^n}{v^n+k^n_H}. \end{aligned}$$

**Fig. 8:**
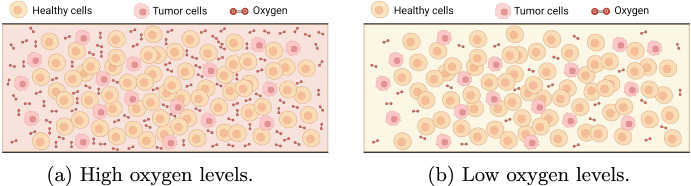
(Color Figure Online) Cell culture model recreating cancer cells in an oxygenated ambient. For illustration purposes, the oxygen is considered as the chemoattractant nutrient of the heterogeneous growth and death. The different local and global oxygen gradients appearing in the chamber may be approximated by approximating Eq. (27b) when considering specific situations, such as high oxygen levels or low oxygen levels. Created with BioRender.com

For any of the above choices, there are numerous possibilities, for instance the cases:

1. There are no other cells at the microfluidic device besides the cell culture of our interest and we are at the low cell regime, $\Pi _4 u/\Pi _3 \ll 1$. In that case, after rapid initial transients describing the diffusion relaxation of the nutrient, and too fast to be on the timescale of cellular motility, Eq. (27b) becomes $v_{xx} = 0$, and the discussion is analogous to the case without the consumption term.
2. There are other non-migrating cells within the microfluidic device in addition to the migrating cells, for instance if we are considering a metastasis model, with the other cells at constant concentration and in excess of the tumour cells. If we additionally have high nutrient concentrations the situation is illustrated in Fig. 8a using oxygen as an example of nutrient. In that case, $w_{\textrm{pc}}(v) \sim 1$ and have
  $$\begin{aligned} w(u,v) = K, \end{aligned}$$
  where *K* is the (dimensionless) total amount of cells, essentially constant as the non-tumour cells are in excess. Then, with the definition
  $$\begin{aligned} \lambda = \Pi _4 K/\Pi _3, \end{aligned}$$
  Equation (27b) becomes
  34 $$\begin{aligned} v_t = \Pi _3 v_{xx} - \Pi _3 \lambda . \end{aligned}$$
  In addition, we assume
  35 $$\begin{aligned} \Pi _3^{-1}\ll \lambda = \frac{\Pi _4 K}{\Pi _3} \ll \Pi _3, \end{aligned}$$
  as motivated in Appendix E, so that $\lambda$ may be treated as order one on using asymptotic methods based on the leading order of approximations based on $\Pi _3\gg 1$. Then the above further reduces to
  36 $$\begin{aligned} v_{xx} = \lambda , \end{aligned}$$
  noting, as above, that fast initial transients are not of interest, with further justification of Eq. (36) in Appendix D via a boundary layer analysis.
3. There are other cells within the microfluidic device at constant concentration and in excess relative to the tumour cells, together with low nutrient concentrations. The situation is also illustrated in Fig. 8b. Assuming the Michaelis-Menten model, $w_\textrm{pc}(v) \sim v/k_m$ so that $w(u,v) = Kv/k_m$ where *K* is the non-dimensional total cell density, effectively constant, and Eq. (27b) reduces to
  37 $$\begin{aligned} v_t = \Pi _3 v_{xx} - \Pi _3 \lambda v, \end{aligned}$$
  where now $\lambda = \Pi _4 K /(k_m\Pi _3)$. As above, this reduces if
  38 $$\begin{aligned} \Pi _3^{-1}\ll \lambda = \frac{\Pi _4 K}{k_m\Pi _3} \ll \Pi _3, \end{aligned}$$
  as assumed with the motivation of Appendix E, in order to yield
  39 $$\begin{aligned} v_{xx} = \lambda v, \end{aligned}$$
  once more noting fast initial transients are not of interest, with additional justification of Eq. (39) presented in Appendix D.

***The presence of other cells in excess and high chemoattractant or oxygen levels***

With the assumptions and motivations as above in the derivation of Eq. (36) we now have

40a $$\begin{aligned} u_t + k \left( v_x u\right) _x&= m(v)u\left( 1-u\right) , \end{aligned}$$

40b $$\begin{aligned} v_{xx}&= \lambda . \end{aligned}$$

where the boundary conditions are again the ones given by Eqs. (26), except for the fact that the flux is given by $f_n=-kv_x u$ as diffusion is neglected and the cell boundary condition at $x=1$, with its associated boundary layer, is no longer considered as discussed in detail previously.

If we prescribe $v(x=0,t) = \psi _1(t)$ and $v(x=1,t) = \psi _2(t)$, Eq. (40b) integrates to

41 $$\begin{aligned} v(x,t)=\frac{1}{2}\lambda x^2 + \left( \psi _2(t) - \psi _1(t) - \frac{1}{2}\lambda \right) x + \psi _1(t). \end{aligned}$$

We have therefore

$$\begin{aligned} \alpha (t,x) = k\lambda x + k\left( \psi _2(t) - \psi _1(t)-\frac{1}{2}\lambda \right) , \end{aligned}$$

and

$$\begin{aligned} \beta (t,x) = m\left( v(t,x)\right) , \end{aligned}$$

for cell growth. Once more, this is a special case of $\alpha (t,x) = a(t)x+b(t)$ with $a(t) = k\lambda$ and $b(t) = k\left( \psi _2(t) - \psi _1(t)-\frac{1}{2}\lambda \right)$, so the different functions needed in order to compute the solution are:

42a $$\begin{aligned} F(t;s)&= s\textrm{e}^{k\lambda t} + \frac{1}{2} \left( 1-\textrm{e}^{k\lambda t}\right) + k\int _0^t\Delta \psi (\eta )\textrm{e}^{-k\lambda \eta } \, \textrm{d}\eta , \end{aligned}$$

42b $$\begin{aligned} G(t;x)&= x\textrm{e}^{-k\lambda t} - \frac{1}{2} \left( \textrm{e}^{-k\lambda t}-1\right) - k\textrm{e}^{-k\lambda t}\int _0^t\Delta \psi (\eta )\textrm{e}^{-k\lambda \eta } \, \textrm{d}\eta , \end{aligned}$$

42c $$\begin{aligned} x^*(t)&= \frac{1}{2} \left( 1-\textrm{e}^{k\lambda t}\right) + k\int _0^t\Delta \psi (\eta )\textrm{e}^{-k\lambda \eta }\, \textrm{d}\eta . \end{aligned}$$

The expression of the cell profile may be computed using Eq. (13), which gives

43 $$\begin{aligned} u(x,t) = \frac{u_0(G(t;x))\textrm{e}^{-k\lambda t}\exp \left( \int _0^t K(\eta , F(\eta ;G(t;x))) \, \textrm{d}\eta \right) }{1 + u_0(G(t;x))\int _0^t K(\eta , F(\eta ;G(t;x)))\textrm{e}^{-k\lambda \eta } \exp \left( \int _0^\eta K(\xi , F(\xi ;G(t;x))) \, \textrm{d}\xi \right) \, \textrm{d}\eta }, \end{aligned}$$

where now

$$\begin{aligned} K(\eta ,X) = m\left( \frac{1}{2}X^2 + (\psi _2(\eta ) - \psi _1(\eta ) - \frac{1}{2}\lambda )X + \psi _1(\eta )\right) . \end{aligned}$$

**Fig. 9:**
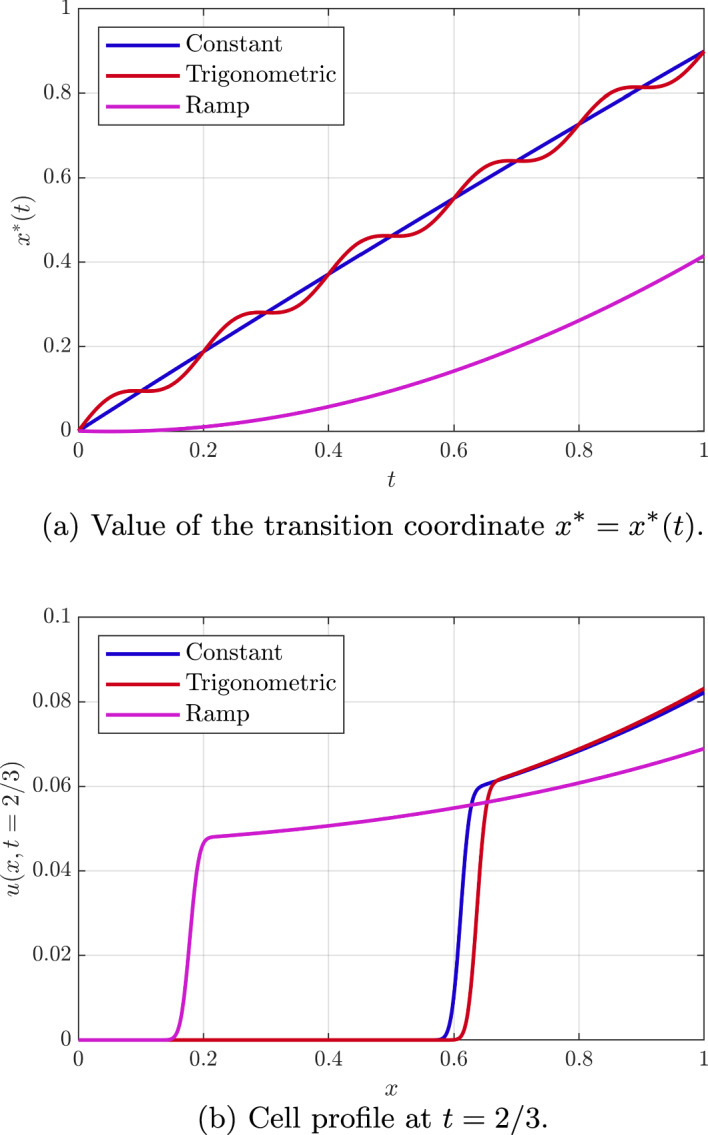
(Color Figure Online) Solution for the case with chemoattractant consumption and high chemoattractant levels. Approximate analytical solution for $k=1$, $m(v)=v$, $\lambda = 0.1$ and the transition region is recreated with $D = 1 \times 10^{-4}$. The initial condition is set again to $u_0(x) = u_0 = 0.05$. We consider three different shapes for $\psi _2$. A constant oxygen level $\psi _2(t) = 1$, an oscillatory stimulus $\psi _2(t) = 1 + \cos (\omega t)$ with $\omega = 10\pi$, and an increasing stimulus $\psi _2(t) = t$

The evolution of the transition coordinate $x^*(t)$ and the dimensionless cell profile for different times are shown in Fig. 9 for $k=1$, $\lambda =0.1$, $m(v) = m_0v$ with $m_0 = 1$, $u_0 = 0.05$ and an external stimulus given by $\psi _1(t) = 0$ and different shapes for $\psi _2(t)$.

While a fast oscillatory stimulus with $\psi _2(t)=1+\cos (10 \pi t)$ plotted in Fig. 9 does not allow a ready approximation for cell density to the right of the transition region, we can consider the cases of $\psi _2(t)=1$ or $\psi _2(t)=t$ that are also considered in this Figure. In particular, we can use elementary but extensive manipulation to determine and approximate

$$\begin{aligned} \int _0^t K(\eta , F(\eta ,s)) \, \textrm{d}\eta \big |_{s=G(t;x)} \end{aligned}$$

to deduce that

$$\begin{aligned} u_0 \approx u_0 \textrm{e}^{-\lambda t} \exp \left( \frac{1-\textrm{e}^{-2\lambda t}}{4\lambda } (x-x^*(t))^2 + \frac{1}{3} h_1(t,\lambda )(x-x^*(t)) + h_2(t,\lambda ) \right) + \mathcal {O}(u_0^2), \end{aligned}$$

in these cases. In particular, $h_1(t,\lambda )$ is of the form

$$\begin{aligned} h_1(t,\lambda ) = \left( 3t + \frac{3}{2} t^2\right) -\left( \frac{3}{2} + \frac{9}{4} t + t^2 \right) \lambda t + \mathcal {O}(\lambda ^2t^2), \end{aligned}$$

for $$\psi _2(t)=1$$ and

$$\begin{aligned} h_1(t,\lambda ) = \left( \frac{3}{2}t^2 + \frac{1}{2}t^3\right) - \left( \frac{3}{2}+ \frac{3}{4}t + \frac{1}{2}t^2+\frac{3}{8}t^3\right) \lambda t + \mathcal {O}(\lambda ^2t^2), \end{aligned}$$

for $$\psi _2(t)=t$$.

For $h_2(t,\lambda )$, we have with $\psi _2(t)=1$ that

$$\begin{aligned} h_2(t, \lambda ) = \frac{1}{2} t^2 \left( 1+\frac{1}{3} t \right) - t \left( \frac{1}{2} + \frac{1}{3} t + \frac{1}{8} t^2 \right) \lambda t + \mathcal {O}(\lambda ^2t^2), \end{aligned}$$

while, in contrast, for $\psi _2(t)=t$ we note that

$$\begin{aligned} h_2(t,\lambda ) = \frac{t^4}{40} \left( 5 + t\right) - t^2\left( \frac{1}{4}+\frac{1}{16}t+\frac{1}{15}t^2+\frac{1}{36}t^3\right) \lambda t + \mathcal {O}(\lambda ^2t^2). \end{aligned}$$

For both cases note that at fixed time the cell concentration to the right of the transition is essentially the exponential of a quadratic in $x-x^*(t)$, provided that $\lambda$ is small enough.

***Other cells and low chemoattractant or oxygen levels.*** With analogous reasoning, Eqs. (27) now reduce to

44a $$\begin{aligned} u_t + k \left( v_x u\right) _x&= m(v)u\left( 1-u\right) , \end{aligned}$$

44b $$\begin{aligned} v_{xx}&= \lambda v. \end{aligned}$$

The general solution to Eq. (44b) generates a chemotactic function $\alpha (t,x)$ that is neither linear in *x* nor separable, with Eq. (6b) equivalent to a Riccati differential equation in $\exp (x)$, for which general solutions are not known in terms of standard functions. Even though the general case is not tractable in terms of constructing solutions, two particular important configurations do allow progress, namely the gradient configuration ($v(x=0,t)=0$ and $v(x=1,t)= \psi (t)$) and the symmetric configuration (($v(x=0,t)=v(x=1,t)=\psi (t)$).

The gradient configuration is certainly the most interesting configuration. For that case, Eq. (44b) integrates to

45 $$\begin{aligned} v(t,x)=\psi (t)\sinh (\sqrt{\lambda } x), \end{aligned}$$

whereby

$$\begin{aligned} \alpha (t,x) = k\sqrt{\lambda }\cosh (\sqrt{\lambda } x)\psi (t), \quad \beta (t,x) = m_0(v(t,x)). \end{aligned}$$

Hence we have separability, with $f(x) = k\sqrt{\lambda }\cosh (\sqrt{\lambda } x)$ and $g(t) = \psi (t)$. With

$$\begin{aligned} {{\mathcal {S}}}(t) = \tan (k\lambda {{\mathcal {T}}}(t) /2), \quad {{\mathcal {T}}}(t) = \int _0^t g(\eta )\textrm{d}\eta , \end{aligned}$$

we in turn have

46a $$\begin{aligned} F(t;s)&= \frac{1}{\sqrt{\lambda }}\ln \left( \frac{1+\textrm{e}^{-s\sqrt{\lambda }}{{\mathcal {S}}}(t)}{\textrm{e}^{-s\sqrt{\lambda }}-{{\mathcal {S}}}(t)}\right) , \end{aligned}$$

46b $$\begin{aligned} G(t;x)&= \frac{1}{\sqrt{\lambda }}\ln \left( \frac{1-\textrm{e}^{- x\sqrt{\lambda }}{{\mathcal {S}}}(t)}{\textrm{e}^{- x\sqrt{\lambda }}+{{\mathcal {S}}}(t)}\right) , \end{aligned}$$

46c $$\begin{aligned} x^*(t)&= \frac{1}{\sqrt{\lambda }}\ln \left( \frac{1+\mathcal{S}(t)}{1-{{\mathcal {S}}}(t)}\right) . \end{aligned}$$

As with the previous cases the expression of the cell profile may be computed using Eq. (13), whereby

47 $$\begin{aligned} u(x,t) = \frac{u_0(G(t;x))\exp \left( \int _0^t K(\eta , F(\eta ;G(t;x))) \, \textrm{d}\eta \right) }{1 + u_0(G(t;x))\int _0^t K(\eta ,F(\eta ;G(t;x))) \exp \left( \int _0^\eta K(\xi , F(\xi ;G(t;x))) \, \textrm{d}\xi \right) \, \textrm{d}\eta },\quad \end{aligned}$$

where $K(\eta ,X) = m(v) - kv_{xx}$, that is

$$\begin{aligned} K(\eta ,X) = m\left( \psi (\eta )\sinh (\sqrt{\lambda } X)\right) - k \lambda \psi (\eta )\sinh (\sqrt{\lambda } X), \end{aligned}$$

and, with $m(v) = m_0 v$ and $u_0(x)=u_0 \ll 1,$ constant, use of the change of variables $X=F(\eta ,s)$ reduces Eq. (47) to

$$\begin{aligned} u(x,t) = u_0 \left( \frac{\cosh (\sqrt{\lambda }G(t;x)) }{\cosh (\sqrt{\lambda } x)} \right) ^{\frac{m_0-k\lambda }{k\lambda } } +\mathcal {O}(u_0^2). \end{aligned}$$

**Fig. 10:**
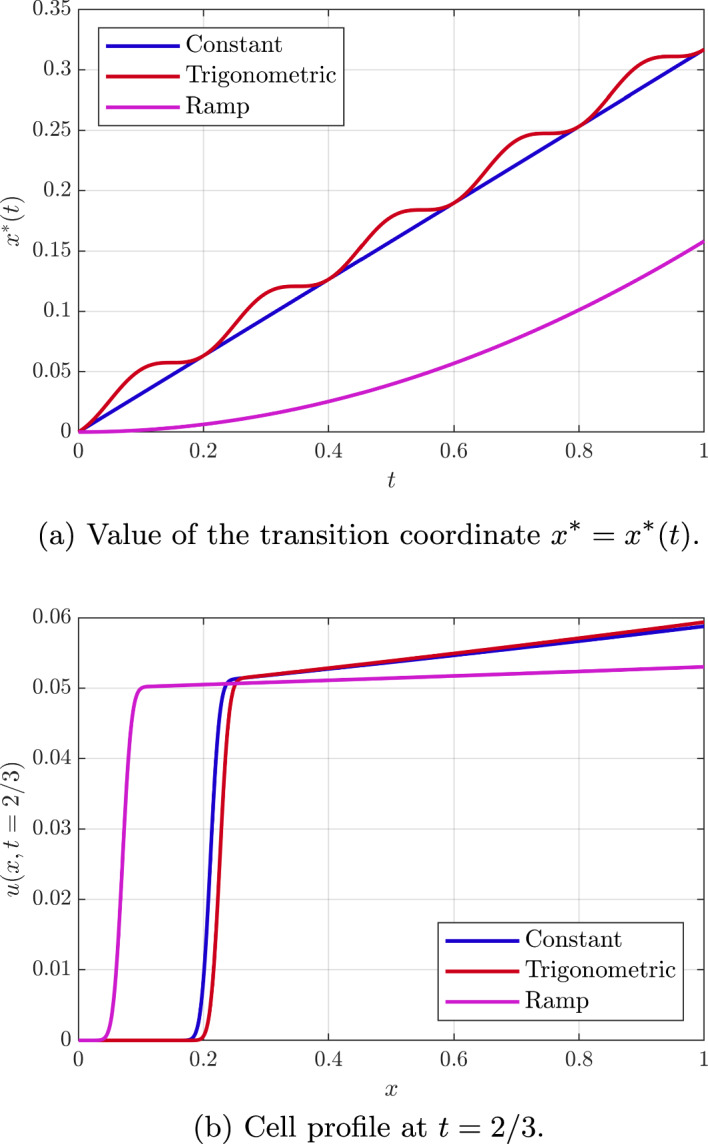
(Color Figure Online) Solution for the case with chemoattractant consumption and low chemoattractant levels. Approximate analytical solution for $k=1$, $m(v) = v$, $\lambda = 0.1$ and the transition region is recreated with $D = 1 \times 10^{-4}$. The initial condition is set again to $u_0(x) = u_0 = 0.05$. We consider three different shapes for $\psi _1$. A constant oxygen level $\psi _1(t) = 1$, an oscillatory stimulus $\psi _1(t) = 1 + \cos (\omega t)$ with $\omega = 10\pi$, and an increasing stimulus $\psi _1(t) = t$

The evolution of the transition coordinate $$x^*(t)$$ and the dimensionless cell profile for different times are shown in Fig. 10 for $$m_0=1$$, $$k=1$$, $$\lambda = 0.1$$, $$ u_0(x)=u_0=0.05,$$ constant. Here, the cellular density to the right of transition further simplifies to

$$\begin{aligned} u(x,t) = u_0 \left( \frac{1+{{\mathcal {S}}}^2(t)}{(1-{{\mathcal {S}}}^2(t)) +2 {{\mathcal {S}}}(t)\sinh \left( \frac{x}{\sqrt{10}}\right) }\right) ^{9} +\mathcal {O}(u_0^2), \quad {{\mathcal {S}}}(t) = \tan \left( \frac{ {{\mathcal {T}}}(t) }{20}\right) , \end{aligned}$$

which entails for small time the spatial variation of the cellular density is that of a hyperbolic sine. Finally, note from the expression for $x^*(t)$ and the fact $x^*(t) \le 1$ for the transition zone to be within the domain, we have the bound ${{\mathcal {T}}} \le \tanh (1/2) < 1$ so that the denominator in the above expression is positive and there is no singularity.

Even if less interesting from the experimental point of view, we can also obtain an expression for a symmetric configuration by considering half of the domain and applying Neumann boundary conditions for $x=0$ so Eq. (44b) is integrated to

48 $$\begin{aligned} v(t,x)=\psi (t)\cosh (\sqrt{\lambda } x). \end{aligned}$$

Again we are under a separable case with $\alpha (t,x) = k\sqrt{\lambda }\sinh (\sqrt{\lambda } x)\psi (t)$ and $\beta (t,x) = 1$, so $f(x) = k\sqrt{\lambda }\sinh (\sqrt{\lambda } x)$ and $g(t) = \psi (t)$, so the different functions needed in order to compute the solution are:

49a $$\begin{aligned} F(t;s)&= \frac{2}{\sqrt{\lambda }} \arctan \left( \textrm{e}^{k\lambda {{\mathcal {T}}}(t) }\tanh \left( \frac{s\sqrt{\lambda }}{2}\right) \right) , \end{aligned}$$

49b $$\begin{aligned} G(t;x)&= \frac{2}{\sqrt{\lambda }} \arctan \left( \textrm{e}^{-k\lambda {{\mathcal {T}}}(t) }\tanh \left( \frac{x\sqrt{\lambda }}{2}\right) \right) , \end{aligned}$$

49c $$\begin{aligned} x^*(t)&= 0. \end{aligned}$$

Now in Eq. (47) we shall use

$$\begin{aligned} K(\eta ,X)= m\left( \psi (\eta )\cosh (\sqrt{\lambda } X\right) - k \lambda \psi (\eta )\cosh (\sqrt{\lambda } X), \end{aligned}$$

and, with $m(v) = m_0 v$ and $u_0(x)=u_0 \ll 1,$ constant, Eq. (47) reduces to

$$\begin{aligned} u(x,t) = u_0 \left( \frac{\sinh (\sqrt{\lambda }x) }{\sinh (\sqrt{\lambda } G(t;x))} \right) ^{\frac{m_0-k\lambda }{k\lambda } } +\mathcal {O}(u_0^2). \end{aligned}$$

## Discussion

There is an increasing use of in vitro investigations in the exploration of cellular motility, for instance with microdevice studies exploring tumour cell dynamics in response to oxygen gradients, as illustrated by glioblastoma studies (Ayuso et al. 2016, 2017; Ayensa-Jiménez et al. 2020). In turn this has motivated the main theme of this paper, namely modelling cell migration chemotaxis in heterogeneous environments, which applies for general chemoattractants, not just oxygen. In particular, the governing equations for cellular motility that have been considered are of the form

$$\begin{aligned} \frac{\partial u}{\partial t} = D \frac{\partial ^2 u}{\partial x^2} - \frac{\partial }{\partial x}\left( \alpha (t,x)u_x\right) + \beta (t,x)u(1-u), \end{aligned}$$

and supplemented by zero flux boundary conditions and suitable, typically constant, initial conditions. Furthermore, the general spatiotemporal heterogeneity in the chemotactic function $\alpha (t,x)$ and the growth function $\beta (t,x)$ emerges from the chemoattractant gradients that the cells respond to, which may be manipulated extensively in microdevice experiments. However, the heterogeneity also entails that while cell migration dynamics towards high concentrations of chemoattractant is anticipated, the dynamics will not simply be that of a translationally invariant travelling wave and their associated analytical simplicity.

Hence, we have considered a framework capable of considering spatiotemporal chemotactic gradients and the resulting wavefront and cell density dynamics for cellular migrations in the presence of spatial and temporal heterogeneity. In particular, the fact cell spreading in the absence of chemotaxis is generally much slower than in its presence, the non-dimensional cellular diffusion coefficient is very small, so that typically $D\ll 1$, which we assume and exploit in this study. With this, we have that away from boundary layers near sources, which were located at the domain edge in the examples considered, and away from cell wavefronts with sharp transitions, an advection equation for the cellular density *u* emerges with $D\sim 0$. Nonetheless, this advection equation is still complex, entailing that a constraint on constructing solutions for the cell density behaviour within the analytical framework presented here is that the chemotactic function $\alpha (t,x)$ must either be linear in *x* or separable with respect to space and time. However, numerous cases are consistent with these constraints, as illustrated by the range of examples considered in Sect. 2.3, together with potential examples that emerge from the consideration of cellular dynamics within microdevices in Sect. 3, as highlighted in Appendix E.

In such contexts, we have illustrated how the method of characteristics can be used to construct the cell density solutions away from boundary layers and transition regions, together with the use of boundary layer methods to construct a uniformly continuous approximate solution that accommodates the transition in the wavefront of the cells. In turn this provides an analytical characterisation of the movement of cellular wavefronts and the concentrations of cells within and either side of the wavefront. However we do not resolve boundary layers near oxygen sources, which has been the righthand boundary in the examples considered, for the presented framework given the limited insight the boundary layer will provide for the overall cell behaviour.

Even with the restriction of linearity or separability of the chemotactic functions $\alpha (t,x)$, there is extensive freedom in the choices of $\alpha (t,x)$ and $\beta (t,x)$. Hence we first considered the predictions of the model for cell behaviour in exemplar test cases. In particular, we have explored and documented the behaviour of the cell density where the chemotactic function varies linearly in space and non-trivially in time, as well exponential spatial behaviour and, in Appendix A, quadratically in space. In addition to analytical investigations, we have also verified that the analytical solutions faithfully reproduce the behaviour of direct numerical solutions of the model, as may be observed extensively in Figs. 3, 4, 5 and 6. Furthermore the exemplar solutions are subsequently used to study models reduced from a full coupling between the cellular density and chemoattractant concentration within a prospective microdevice setting, as documented in Sect. 3. Once more the resulting dynamics are analytically investigated and documented, with rationally based analytical approximation for the location of the transition region and the cellular density presented.

Such analytical solutions are useful in numerous ways. For example, they provide oversight and insight for the system dynamics across parameter space. To illustrate, we consider the wavefront being driven by chemoattractant at the right-hand boundary, so that $\psi _1(t)=0, v(t,1)=\psi _2(t)$. Then, with *k* a measure of the strength of chemotaxis, differentiating $x^*(t)$ from Eq. (42c) for the speed for the transition front gives

$$\begin{aligned} \frac{\textrm{d}x^*}{\textrm{d}t}= -\frac{1}{2} k\lambda \textrm{e}^{k\lambda t} + k \psi _2(t)\textrm{e}^{-k\lambda t} \end{aligned}$$

for the model of Sect. 3.1.2, with high chemoattractant or oxygen levels. Hence, in this case, increasing the chemoattractant/oxygen uptake, as representing by increasing $\lambda$, always slows the propagation of a rightmoving wave. In contrast, for the model with low chemoattractant or oxygen levels of Sect. 3.1.2 differentiating $x^*(t)$ from Eq. (46c) we obtain

$$\begin{aligned} \frac{\textrm{d}x^*}{\textrm{d}t}= \frac{\sqrt{\lambda }}{\textrm{cos}\left( k\lambda \mathcal {T}\right) }\psi _2(t) \end{aligned}$$

therefore revealing that increasing the chemoattractant/oxygen uptake always speeds up the propagation of the rightmoving wave, illustrating the general deductions that may be made from the presented analytical solutions.

As well as providing analytical characterisation of the systems behaviour the analytical approximations provide a means to very rapidly compute cell behaviour. Thus the approximate analytical solutions can support computationally intensive studies. A simple example would be a global sensitivity analyses over all parameters. In particular, rapid evaluation would be most useful for parameter estimation using experimental, often noisy, data especially if Bayesian techniques are considered as this require extensive simulation to provide posterior distributions, rather than optimisation techniques which only generate point estimates for parameters.

A directly analogous example is Bayesian model selection, whereby the comparison of experimental data and model prediction is used to distinguish different model structures when these are not known a priori, such as different functional forms of $\alpha (t,x)$ or $\beta (t,x)$ representing different prospective growth and chemotactic responses for a given the tumour cell line in question. In particular both optimisation techniques and the Bayesian techniques are iterative, so that the use of a rational but rapid evaluation of an approximation to an optimum in optimisation studies or a posterior for Bayesian techniques can in turn be used to restart the procedure with the full numerical model to further refine the results (Brown et al. 2022). As well as investigating the prospective role of such analytical solutions in parameter estimation, future work might also contemplate the analytical structure and solution for problems in higher spatial dimension, though it is unclear at this stage how tractable such a study would be.

In summary, we have developed a framework for the construction of analytical approximations for front behaviours and densities for cells undergoing chemotaxis in heterogeneous environments, as characterised by the chemotactic and growth functions $\alpha$ and $\beta$ respectively. The resulting cellular waves of migration are not simple travelling waves due to the heterogeneity induced by the chemoattractant profiles. Nonetheless, numerous features of the wavefronts have been extracted via rational approximation, such as the location and speed of the propagating wave, together with the cellular density profile. These have been explored and validated for exemplar scenarios as well as investigated for models fundamentally motivated by prospective experimental microdevices for observing cellular motility under a very wide range of conditions, even if complete generality is not feasible for progress using constructive methods. Thus, the solutions presented here not only provide insight into the behaviour of cellular motility under the influence of spatiotemporal chemotactic heterogeneity, but also highlight general behaviours and important mechanisms, as well as providing a means of rapid evaluation in demanding computational studies, such as Bayesian parameter estimation and model selection.

## Figures and Tables

**Fig. 11:**
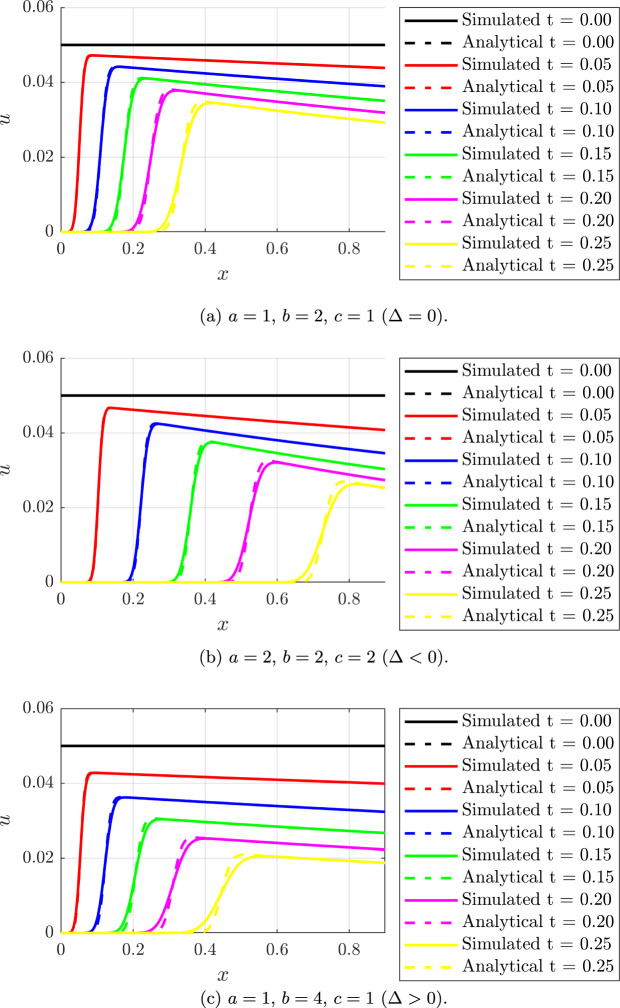
(Color Figure Online) Comparison of numerical and analytical solutions for $\alpha$ quadratic. The analytical and simulated (for $D = 1 \times 10^{-3}$) profiles at different times are compared for three different $\alpha (t,x) = ax^2+bx+c$ expressions

## Data Availability

Data sharing not applicable to this article as no datasets were generated or analysed during the current study.

## Appendix A: Quadratic Chemotaxis

As an extension to the linear and exponential chemotaxis cases, we now consider a function of the form

$$\begin{aligned} \alpha (t,x) = (ax^2+bx+c)g(t), \end{aligned}$$

and hence $$f(x)=ax^2+bx+c$$.

- If $\Delta = b^2-4ac = 0$, Eq. (9) gives
  $$\begin{aligned} \frac{1}{2as+b}-\frac{1}{2ax+b} = \frac{1}{2}\int _0^\tau g(\eta )\,\textrm{d}\eta = \frac{1}{2} {{\mathcal {T}}}(\tau ), \end{aligned}$$
  and in turn
  $$\begin{aligned} F(t;s)&= \frac{4as+2abs {{\mathcal {T}}}(t) +b^2 {{\mathcal {T}}}(t) }{4a-2ba {{\mathcal {T}}}(t) -4a^2s {{\mathcal {T}}}(t) }, \\ G(t;x)&= \frac{4ax-2ab {{\mathcal {T}}}(t) x-b^2 {{\mathcal {T}}}(t) }{4a^2 x {{\mathcal {T}}}(t) +2ab {{\mathcal {T}}}(t) +4a}. \end{aligned}$$
  The transition is located at
  $$\begin{aligned} x^*(t) = \frac{b^2 {{\mathcal {T}}}(t) }{4a-2ba {{\mathcal {T}}}(t) } = \frac{c {{\mathcal {T}}}(t) }{1-b {{\mathcal {T}}}(t) /2}. \end{aligned}$$
  Furthermore, in the case where $g(t)=\beta (t,x)=1$ we have to an accuracy of $\mathcal {O}(u_0^2)$ that
  $$\begin{aligned} u(x,t) = u_0 \textrm{e}^t \frac{f(s)}{f(x)} = u_0 \textrm{e}^t \frac{as^2+bs+c}{ax^2+bx+c}\bigg |_{s=G(t;x)}= \frac{4u_0\textrm{e}^t}{(2+bt +2atx)^2}, \end{aligned}$$
  via use of Eq. (15), with $u_0\ll 1$ where significant, but elementary, manipulation is required to deduce the final expression. In particular for the parameters of Fig. 11a it is straightforward to show that at leading order the behaviour in *x* for *t* fixed is linear, as observed in this figure.
- If $\Delta = b^2-4ac < 0$, Eq. (9) yields
  $$\begin{aligned} \left( \arctan \left( \frac{2ax+b}{\sqrt{ -\Delta }}\right) -\arctan \left( \frac{2as+b}{\sqrt{ -\Delta }}\right) \right) = \frac{1}{2}\sqrt{ -\Delta }\int _0^\tau g(\eta )\,\textrm{d}\eta = \frac{1}{2}\sqrt{ -\Delta }{{\mathcal {T}}}(\tau ), \end{aligned}$$
  so that
  $$\begin{aligned} F(t;s)&= \frac{1}{2a}\left[ \sqrt{-\Delta }\tan \left( \frac{1}{2}\sqrt{-\Delta } {{\mathcal {T}}}(t) +\arctan \frac{2as+b}{\sqrt{-\Delta }}\right) -b\right] , \\ G(t;x)&= \frac{1}{2a}\left[ \sqrt{-\Delta }\tan \left( -\frac{1}{2}\sqrt{-\Delta } {{\mathcal {T}}}(t) +\arctan \frac{2ax+b}{\sqrt{-\Delta }}\right) -b\right] , \end{aligned}$$
  with the transition is located at
  $$\begin{aligned} x^*(t) = \frac{1}{2a}\left[ \sqrt{-\Delta }\tan \left( \frac{1}{2}\sqrt{-\Delta } {{\mathcal {T}}}(t) +\arctan \frac{b}{\sqrt{-\Delta }}\right) -b\right] . \end{aligned}$$
  Furthermore, the analytical and full numeric solutions are plotted for the parameters $a=b=c=2$ with $\beta (t,x)=g(t)=1$ in Fig. 11b. For this set of parameters, where $t\le 0.25$ as in Fig. 11b, we have
  $$\begin{aligned} \tan \left( \frac{t\sqrt{-\Delta }}{2}\right) \approx \frac{t\sqrt{-\Delta }}{2} \left( 1+ O\left( \frac{1}{3} \left( \frac{t\sqrt{-\Delta }}{2} \right) ^2\right) \right) \approx \frac{t\sqrt{-\Delta }}{2}, \end{aligned}$$
  noting
  $$\begin{aligned} \frac{1}{3} \left( \frac{t\sqrt{-\Delta }}{2} \right) ^2 \lesssim 0.06. \end{aligned}$$
  Dropping the corrections in higher powers of $t\sqrt{-\Delta }$, valid for sufficiently small time including the times plotted in Fig. 11, we have
  $$\begin{aligned} x^*(t) = \frac{ct}{1-bt/2}, \end{aligned}$$
  and then also dropping terms scaling with $\mathcal {O}(u_0^2)$, one finds
  $$\begin{aligned} u(x,t) = u_0 \textrm{e}^t \frac{f(s)}{f(x)} = u_0 \textrm{e}^t \frac{as^2+bs+c}{ax^2+bx+c}\bigg |_{s=G(t;x)}= \frac{u_0\textrm{e}^t (4-t^2\Delta ) }{(2+bt +2atx)^2}, \end{aligned}$$
  with a relative correction of $(1+\mathcal {O}([t\sqrt{-\Delta }/2] ^2/3)).$ The latter again gives an approximate linear dependence in *x* for *t* fixed given the parameters of Fig. 11b, as observed.
- If $\Delta = b^2-4ac > 0$, Eq. (9) gives
  $$\begin{aligned} \ln \left( \frac{2ax+b-\sqrt{\Delta }}{2ax+b+\sqrt{\Delta }}\right) -\ln \left( \frac{2as+b-\sqrt{\Delta }}{2as+b+\sqrt{\Delta }}\right) = \sqrt{\Delta }\int _0^\tau g(\eta )\,\textrm{d}\eta = \sqrt{\Delta }\mathcal {T}(\tau ), \end{aligned}$$
  so that
  $$\begin{aligned} F(t;s)&= \frac{1}{2a}\left[ \frac{\gamma ^+(2as + \gamma ^-)\exp (\frac{1}{2}\sqrt{\Delta } {{\mathcal {T}}}(t) ) - \gamma ^-(2as + \gamma ^+)\exp (-\frac{1}{2}\sqrt{\Delta } {{\mathcal {T}}}(t) )}{(2as+\gamma ^+)\exp (-\frac{1}{2}\sqrt{\Delta } {{\mathcal {T}}}(t) ) - (2as+\gamma ^-)\exp (\frac{1}{2}\sqrt{\Delta } {{\mathcal {T}}}(t) )}\right] , \\ G(t;x)&= \frac{1}{2a}\left[ \frac{\gamma ^+(2ax + \gamma ^-)\exp (-\frac{1}{2}\sqrt{\Delta } {{\mathcal {T}}}(t) ) - \gamma ^-(2ax + \gamma ^+)\exp (\frac{1}{2}\sqrt{\Delta } {{\mathcal {T}}}(t) )}{(2ax+\gamma ^+)\exp (\frac{1}{2}\sqrt{\Delta } {{\mathcal {T}}}(t) ) - (2ax+\gamma ^-)\exp (-\frac{1}{2}\sqrt{\Delta } {{\mathcal {T}}}(t) )}\right] , \end{aligned}$$
  where we have defined $\gamma ^+ = b+\sqrt{\Delta }$ and $\gamma ^- = b-\sqrt{\Delta }$, with the transition location given by
  $$\begin{aligned} x^*(t) = 2c\left[ \frac{\exp (\frac{1}{2}\sqrt{\Delta } {{\mathcal {T}}}(t) ) - \exp (-\frac{1}{2}\sqrt{\Delta } {{\mathcal {T}}}(t) )}{\gamma ^+\exp ( -\frac{1}{2}\sqrt{\Delta } {{\mathcal {T}}}(t) ) - \gamma ^-\exp ( \frac{1}{2}\sqrt{\Delta } {{\mathcal {T}}}(t) })\right] . \end{aligned}$$
  For Fig. 11c, we again have $\beta (t,x)=1=g(t)$, so that ${{\mathcal {T}}}(t)=t$; we also have $t\sqrt{\Delta }/2 \ll 1$ throughout the simulation regime. Hence, on neglecting higher powers of $t\sqrt{-\Delta }/2$, the transition location simplifies to
  $$\begin{aligned} x^*(t) = \frac{ct}{1- bt/2 }, \end{aligned}$$
  which agrees with the above as $t\sqrt{-\Delta } / 2 \rightarrow 0.$ Furthermore, under these conditions with $u_0^2\ll 1$ and neglecting higher orders in $u_0$, one finds
  $$\begin{aligned} u(x,t) = u_0 \textrm{e}^t \frac{f(s)}{f(x)} = u_0 \textrm{e}^t \frac{as^2+bs+c}{ax^2+bx+c}\bigg |_{s=G(t;x)}= u_0 \textrm{e}^t(1-(b+2ax)t), \end{aligned}$$
  with an relative error of $(1+\mathcal {O}([t\sqrt{-\Delta }/2]^2)).$ For *t* fixed, again we have *u*(*x*, *t*) is approximately linearly decreasing in *x*, as observed in Fig. 11c.

More generally, Fig. 11 shows a comparison between the numerical results with $D=1\times 10^{-3}$, $\beta (t,x)=1=g(t)$ and the analytical solution for three different expressions of $\alpha (x)$ and $\beta (t,x) = 1$, with full consistency with the above solutions and approximations.

## Appendix B: Oscillatory Solutions for Homogeneous Growth

We consider homogeneous growth, so that $\beta (t,x)$ is a constant independent of *x* and *t*, together with a temporally oscillating chemotactic response governed by $\alpha (t,x)$, so that Eq. (19) reduces to

50 $$\begin{aligned} r'+\left( \beta -a\cos (\omega t)\right) r =\beta , \quad r(0) = r^*. \end{aligned}$$

Hence the cell concentration to the right of the transition region, but away from the boundary layer at $x=1$, is spatially constant with a temporal oscillation, which we consider below for four distinct parameter regimes.

### B.1 Slow Variations of the Gradients, ${\varvec{\omega \ll 1}}$

Let $T = \omega t$ and, without loss of generality, we consider the decomposition $r(t) = y+z(\omega t)$. Then, Eq. (50) becomes

51 $$\begin{aligned} \omega z'(\omega t) + \frac{\textrm{d}y}{\textrm{d}t} + \left( \beta - a\cos (\omega t)\right) z(\omega t) + \left( \beta - a\cos (\omega t)\right) y = \beta . \end{aligned}$$

Further, let $\omega z'(\omega t) + \left( \beta - a\cos (\omega t)\right) z(\omega t)=\beta$, so we have

52 $$\begin{aligned} \omega z'(T) +\left( \beta - a\cos (T)\right) z(T) = \beta . \end{aligned}$$

Since $\omega \ll 1$, we can approximate the solution of Eq. (52) by

53 $$\begin{aligned} z(T) = \frac{\beta }{\beta -a\cos (T)} + \mathcal {O}(\omega ). \end{aligned}$$

Then, Eq. (51) yields

54 $$\begin{aligned} \frac{\textrm{d}y}{\textrm{d}t}+ \left( \beta - a\cos (\omega t)\right) y = 0, \end{aligned}$$

and the initial condition is

55 $$\begin{aligned} y^* = y(t=0) = r(t=0) - z(0) = r^* - \frac{\beta }{\beta - a}. \end{aligned}$$

As we have a slow modulation of the frequency/decay rate, we use the Wentzel-Kramers-Brillouin (WKB) method (Olver 1997). In terms of $T = \omega t$, Eq. (54) becomes

56 $$\begin{aligned} \omega \frac{\textrm{d}y}{\textrm{d}T} + \left( \beta - a\cos (T)\right) y = 0, \quad y(0) = y^*. \end{aligned}$$

The WKB approximation is expressed here as

57 $$\begin{aligned} y = p\exp \left( \frac{\phi (T)}{\omega }\right) J(T), \quad J(T) = J_0 + \omega J_1 + \mathcal {O}(\omega ^2). \end{aligned}$$

Substituting Eq. (57) into Eq. (54) we obtain

58 $$\begin{aligned} p\exp \left( \frac{\phi (T)}{\omega }\right) \left[ \omega \left( \frac{\dot{\phi }}{\omega }J + {\dot{J}}\right) +(\beta - a\cos T)J\right] = 0. \end{aligned}$$

Therefore, $\omega \left( \frac{\dot{\phi }}{\omega }J + {\dot{J}}\right) +(\beta - a\cos T)J=0$, so that

59 $$\begin{aligned} \dot{\phi }\left( J_0 + \omega J_1 + \ldots \right) + \omega \left( {\dot{J}}_0 + \omega {\dot{J}}_1 + \ldots \right) + \left( \beta - a\cos T\right) \left( J_0 + \omega J_1 + \ldots \right) = 0.\nonumber \\ \end{aligned}$$

The $\mathcal {O}(1)$ corresponding equation is

60 $$\begin{aligned} J_0\left( \dot{\phi } + (\beta - a \cos T)\right) = 0, \end{aligned}$$

and solving it for $\phi$ gives

61 $$\begin{aligned} \phi (T) = \phi ^* -\beta T + a \sin T. \end{aligned}$$

The $\mathcal {O}(\omega )$ corresponding equation is

62 $$\begin{aligned} J_1\left( \dot{\phi } + (\beta - a \cos T)\right) + {\dot{J}}_0 = 0, \end{aligned}$$

so, as $\dot{\phi } + (\beta - a \cos T)=0$, we obtain $J_0 = J_0^*$ (constant). Consequently, Eq. (57) becomes

63 $$\begin{aligned} y = K\exp \left( \frac{-\beta T+a\sin T}{\omega }\right) , \end{aligned}$$

for $K = p\exp (\frac{\phi ^*}{\omega })J_0$ constant. Using that $T = \omega t$ and the initial value $y(0) = y^*$, we obtain the approximation

64 $$\begin{aligned} y = \left( r^* - \frac{\beta }{\beta - a} \right) \exp \left( -\beta t +\frac{a}{\omega }\sin (\omega t)\right) . \end{aligned}$$

Finally, as $r = y + z(\omega t)$, we have

65 $$\begin{aligned} r \sim \frac{\beta }{\beta -a\cos (\omega t)} + \left( r^* - \frac{\beta }{\beta - a} \right) \exp \left( -\beta t +\frac{a}{\omega }\sin (\omega t)\right) . \end{aligned}$$

### B.2 Fast Variations of the Gradients, ${\varvec{\omega \gg 1}}$

We solve now the problem using the method of multiple scales. Let us assume that $r(t) = r(T_1,T_2)$, where $T_1 = t$ and $T_2 = \omega t$, so that

66 $$\begin{aligned} \frac{dr}{dt} = \frac{\partial r}{\partial T_1}\frac{\partial T_1}{\partial t} + \frac{\partial r}{\partial T_2}\frac{\partial T_2}{\partial t} = \frac{\partial r}{\partial T_1} + \omega \frac{\partial r}{\partial T_2}. \end{aligned}$$

If $\varepsilon = \frac{1}{\omega } \ll 1$, Eq. (50) becomes

67 $$\begin{aligned} \varepsilon \left( \frac{\partial r}{\partial T_1} + \frac{\partial r}{\partial T_2}\right) + \varepsilon \left( \beta - a\cos (T_2)\right) r= \varepsilon \beta . \end{aligned}$$

If we use an asymptotic expansion of *r*, $r = r_0 +\varepsilon r_1$ we obtain:

68 $$\begin{aligned} \varepsilon \left( \frac{\partial r_0}{\partial T_1} + \varepsilon \frac{\partial r_1}{\partial T_1}\right) + \frac{\partial r_0}{\partial T_2} + \varepsilon \frac{\partial r_1}{\partial T_2} + \varepsilon \left( \beta - a\cos (T_2)\right) \left( r_0 + \varepsilon r_1\right) + \mathcal {O}(\varepsilon ^2) = \varepsilon \beta .\nonumber \\ \end{aligned}$$

Solving the equation obtained collecting the $\mathcal {O}(1)$ terms, we find $r_0$ is a function of $T_1$ only, that is

69 $$\begin{aligned} r_0 = r_0(T_1), \end{aligned}$$

with the initial condition $r_0(0) = r^*.$ Now, for the equation obtained collecting the $\mathcal {O}(\varepsilon )$ terms, we have

70 $$\begin{aligned} r_0'(T_1) + \frac{\partial r_1}{\partial T_2} + (\beta - a\cos (T_2))r_0(T_1) = \beta . \end{aligned}$$

As $r_1$ is a periodic correction, integrating Eq. (70) over $T_2\in [0,2\pi ]$ we obtain

71 $$\begin{aligned} 2\pi r_0'(T_1) + 2\pi \beta r_0(T_1) = 2\pi \beta , \end{aligned}$$

and thus, solving for $r_0(T_1)$, we have

72 $$\begin{aligned} r_0(T_1) = r^*e^{-\beta T_1} + (1-e^{-\beta T_1}). \end{aligned}$$

Hence, the leading order approximation is

73 $$\begin{aligned} r(t) \sim r^*e^{-\beta t} + (1-e^{-\beta t}). \end{aligned}$$

Now, for the $\mathcal {O}(\varepsilon )$ equation we have:

74 $$\begin{aligned} \frac{\partial r_1}{\partial T_2} = a\cos (T_2)r_0(T_1). \end{aligned}$$

Since $$r_1(T_1,T_2 = 0) = 0$$,

75 $$\begin{aligned} r_1(T_1,T_2) = a\sin (T_2)r_0(T_1). \end{aligned}$$

and therefore the first order correction is, $r_0 + \varepsilon r_1$, that is:

76 $$\begin{aligned} r(t) \sim \left( r^*e^{-\beta t} + (1-e^{-\beta t})\right) \left( 1 + \frac{a}{\omega }\sin (\omega t)\right) . \end{aligned}$$

### B.3 Dominant Chemotaxis, ${\varvec{a\gg \beta .}}$

We solve now the problem using the standard asymptotic expansion method. We set $\varepsilon = \beta /a \ll 1$. Then, Eq. (50) reduces to

77 $$\begin{aligned} r' + a\left( \varepsilon -\cos (\omega t)\right) r = a\varepsilon . \end{aligned}$$

The leading order solution is obtained immediately as it is the solution to the homogeneous linear differential equation:

78 $$\begin{aligned} r_0'-a\cos (\omega t)r_0=0. \end{aligned}$$

Thus the leading order approximation is

79 $$\begin{aligned} r\sim r_0=r^*e^{\frac{1}{\omega }a\sin (\omega t)}, \end{aligned}$$

though the next order correction generates a cumbersome expression and thus is not presented.

### B.4 Dominant Cell Proliferation, ${\varvec{a\ll \beta .}}$

Again, we solve the problem using the standard asymptotic expansion method. Now, we set $\varepsilon = a/\beta \ll 1$. Then, Eq. (50) gives

80 $$\begin{aligned} r' + \beta \left( 1-\varepsilon \cos (\omega t)\right) r = \beta . \end{aligned}$$

The leading order solution is obtained immediately as the solution to the inhomogeneous linear differential equation

81 $$\begin{aligned} r_0'+\beta r_0=\beta , \end{aligned}$$

and hence is given by

82 $$\begin{aligned} r \sim r_0=r^*e^{-\beta t} + \left( 1-e^{-\beta t}\right) . \end{aligned}$$

For the first correction, an asymptotic expansion of *r* of the form $r=r_0 + \varepsilon r_1$ reveals that

83 $$\begin{aligned} r_1'+\beta r_1=\beta \cos (\omega t)r_0. \end{aligned}$$

The solution to this ODE with initial condition $r_1(0) = 0$ is given by

84 $$\begin{aligned} r_1(t) = \frac{\beta \left[ (r^*-1)\gamma ^2\sin (\omega t)+\omega ^2e^{\beta t}\sin (\omega t) + \omega \beta e^{\beta t}\cos (\omega t)\right] }{\omega \gamma ^2e^{\beta t}}-\frac{\beta ^2}{\gamma ^2}e^{-\beta t},\qquad \end{aligned}$$

where we have defined $\gamma ^2 = \beta ^2+\omega ^2$. Hence up to the first order correction we have

85 $$\begin{aligned}{} & {} r(t) \sim r_0 + \varepsilon r_1 = \nonumber \\{} & {} \quad 1+\frac{(r^*-1)\gamma ^2 \omega -A\left[ \left( \gamma ^2r^*-\gamma +\omega ^2e^{\beta t}\right) \sin (\omega t)+ \omega \beta e^{\beta t}\cos (\omega t)-\beta \omega \right] }{\omega \gamma ^2 e^{\beta t}}.\nonumber \\ \end{aligned}$$

## Appendix C: Error Indicators

For reference, we present select error indicators for the different approximations presented.

### C.1 Errors in Space

For the approximations presented in Figs. 3, 4, 6 and 11, the error is estimated using the $L_2$ norm in [0, 0.8]. The maximum of the interval is set to 0.8 to avoid the influence of the right boundary layer, while the boundary layer at $x=0$ is only relevant for small time and not for the times considered below in the error indicator tables. With a normalisation of the domain length, we therefore define

86 $$\begin{aligned} \textrm{E}(t) = \frac{1}{\sqrt{0.8}}\sqrt{\int _0^{0.8} \left( {\hat{u}}(x,t) - u(x,t)\right) ^2 \, \textrm{d}x}, \end{aligned}$$

where *u* is the numerical solution and ${\hat{u}}$ the approximate composite solution obtained using the analytical expressions. Table 1 presents the $L_2$ errors for the linear chemotaxis case, Table 2 for the exponential chemotaxis and Table 3 for the quadratic chemotaxis case.

**Table 1:** Error indicators for the linear chemotaxis case. The error is computed for numerous times, corresponding to those presented in Figs. 3 and 4

*t*	Constant gradient	Oscillatory gradient
0.04	$0.08 \times 10^{-3}$	$0.08 \times 10^{-3}$
0.09	$0.22 \times 10^{-3}$	$0.11 \times 10^{-3}$
0.14	$0.37 \times 10^{-3}$	$0.12 \times 10^{-3}$
0.19	$0.53 \times 10^{-3}$	$0.09 \times 10^{-3}$
0.25	$0.69 \times 10^{-3}$	$0.32 \times 10^{-3}$

**Table 2:** Error indicators for the exponential chemotaxis case. The error is computed for numerous times, corresponding to those presented in Fig. 6

*t*	Exponential function
0.04	$0.10 \times 10^{-3}$
0.09	$0.25 \times 10^{-3}$
0.14	$0.44\times 10^{-3}$
0.19	$0.67 \times 10^{-3}$
0.25	$1.00 \times 10^{-3}$

**Table 3:** Error indicators for the quadratic case. The error is computed for numerous times, corresponding to those presented in Fig. 11

*t*	$\Delta = 0$	$\Delta < 0$	$\Delta > 0$
0.04	$0.09 \times 10^{-3}$	$0.12 \times 10^{-3}$	$0.17 \times 10^{-3}$
0.09	$0.24 \times 10^{-3}$	$0.29 \times 10^{-3}$	$0.41 \times 10^{-3}$
0.14	$0.41 \times 10^{-3}$	$0.52 \times 10^{-3}$	$0.64 \times 10^{-3}$
0.19	$0.59 \times 10^{-3}$	$0.77 \times 10^{-3}$	$0.84 \times 10^{-3}$
0.25	$0.79 \times 10^{-3}$	$1.02 \times 10^{-3}$	$1.01 \times 10^{-3}$

### C.2 Errors in Time

For comparison purposes, we compute the approximation error for the solutions obtained using asymptotic theory of Fig. 5. With a normalisation of the domain length this is defined as

87 $$\begin{aligned} \textrm{E}_i = \frac{1}{\sqrt{50}}\sqrt{\int _0^{50} \left( {\hat{u}}_i^*(t) - u^*(t)\right) ^2 \, \textrm{d}t}, \end{aligned}$$

where ${\hat{u}}^*_i$ is the approximated solution of order *i* and $u^*$ is the numerical solution using standard Runge–Kutta solvers, with the results presented in Table 4 for the different approximations.

**Table 4:** Error indicators for the linear polynomial case. The error is computed in the interval [0, 50] for the four different cases analysed in Fig. 5 and both orders of approximation

Case	Order 0	Order 1
$\omega \ll 1$	0.053	0.018
$\omega \gg 1$	0.068	0.012
$\beta \ll a$	0.002	–
$a \ll \beta$	0.048	0.003

## Appendix D: Simplification of the Transport Equation for Chemotaxis

With *v*(*t*, *x*) denoting the concentration of the chemoattractant, and with $\Pi _3\gg 1$ we have in Sect. 3.1 an equation of the form

$$\begin{aligned} v_t = \Pi _3 v_{xx}, \end{aligned}$$

since $w(u,v) =0$ has been imposed on (27b), while in Sect. 3.1.2 we have equations of one of the two forms

$$\begin{aligned} v_t = \Pi _3 v_{xx} - \Pi _3\lambda , \qquad v_t = \Pi _3 v_{xx} -\Pi _3 \lambda v, \end{aligned}$$

via Eqs. (34), (37) respectively. These are accompanied by boundary conditions of the form

$$\begin{aligned} v(t,0)= \psi _1(t), \quad v(t,1) = \psi _2(t), \end{aligned}$$

and initial conditions $v(0,x)=v_0(x)$ are required to close the system. For simplicity, we assume the boundary conditions and initial conditions are consistent at $(t,x)=(0,0), (0,1)$.

To further proceed we firstly assume $\Pi _3^{-1}\ll \lambda \ll \Pi _3$, so that $\lambda$ can be treated as unit order of magnitude in asymptotic methods based on $\varepsilon = \Pi _3^{-1}\ll 1$, and we also assume that $\psi _1(t),$ $\psi _2(t)$ have derivatives that are unit order of magnitude, or less. With these weak assumptions, our objective is show that the time derivative $v_t$ can be neglected at leading order, justifying the use of Eqs. (28b), (36), (39) in the main text and also justifying the neglect of the consideration of initial conditions in the main text on the grounds this only governs fast initial transients.

Below we work with the PDE

88 $$\begin{aligned} v_t = \Pi _3 v_{xx} - \mu \Pi _3 \lambda v^\zeta , \end{aligned}$$

with $\mu ,~\zeta \in \{0,1\}$ so that $\mu =0$ gives one the above equations while $\mu =1,~\zeta =0$, gives another with the final possibility corresponding to $\mu =1,~\zeta =1$, allowing the three cases to be considered simultaneously.

We have an outer timescale of *t* and an inner timescale of $\tau = \Pi _3 t=t/\varepsilon$. In the outer region, $t\gg \epsilon$, for the leading order outer solution $v^\textrm{out}(t,x)$ one indeed has

$$\begin{aligned} 0 = v^\textrm{out}_{xx} - \mu \lambda (v^\textrm{out})^\zeta , \quad v^\textrm{out}(t,0)= \psi _1(t), \quad v^\textrm{out}(t,1) = \psi _2(t), \end{aligned}$$

without consideration of the initial condition. Hence the solutions presented in the main text, for instance Eqs. (29), (41), (45), (48), are the same as the leading order outer solutions. With the leading order inner solution $v^\textrm{in}(\tau ,x)$ we have

$$\begin{aligned} v^\textrm{in}_\tau = v^\textrm{in}_{xx} - \mu \lambda ( v^\textrm{in})^\zeta , \quad v^\textrm{in}(0,x)=v_0(x), \end{aligned}$$

and the leading order boundary conditions

$$\begin{aligned} v^\textrm{in}(\tau ,0)= \psi _1(\varepsilon \tau ) = \psi _1(0), \quad v^\textrm{in}(\tau ,1)= \psi _2(\varepsilon \tau ) = \psi _2(0), \end{aligned}$$

on noting $t=\varepsilon \tau$ and where $\mathcal {O}(\varepsilon )$ corrections are dropped in the final term for both boundary conditions. Recalling $\mu ,~\zeta \in \{0,1\}$ and using the decomposition

$$\begin{aligned} v^\textrm{in}(\tau ,x) = v^\textrm{out}(0,x) + q(\tau ,x), \end{aligned}$$

without loss of generality, we have

$$\begin{aligned} q_\tau = q_{xx} -\mu \zeta \lambda q, \quad q(\tau ,0)=q(\tau ,1)=0, \quad q(0,x) = v_0(x)-v^\textrm{out}(0,x). \end{aligned}$$

With the Fourier decomposition of $q(\tau ,x)$ and its initial condition

$$\begin{aligned} q(x,\tau ) = \sum _{n=1}^\infty q_n(\tau ) \sin \left( n\pi x \right) , \quad q(0,x) = \sum _{n=1}^\infty q_n^0 \sin \left( n\pi x \right) , \end{aligned}$$

we have

$$\begin{aligned} \frac{\textrm{d}q_n}{\textrm{d}\tau } = -n^2\pi ^2 q_n - \mu \zeta \lambda q_n, \quad q_n(0) = q_n^0, \end{aligned}$$

so that

$$\begin{aligned} q(x,\tau ) = \sum _{n=1}^\infty q_n^0 \sin \left( n\pi x \right) \textrm{e}^{-(n^2\pi ^2+\mu \zeta \lambda )\tau } \approx 0 \quad \text{ for } \quad \pi ^2 \tau \gg 1. \end{aligned}$$

Thus the leading order composite solution is given by

89 $$\begin{aligned} v(t,x)= & {} v^\textrm{out}(t,x) + v^\textrm{in}(\tau (t),x) - \lim _{t \rightarrow 0}v^\textrm{out}(t,x) =v^\textrm{out}(t,x) + q(\tau (t),x) \nonumber \\\approx & {} v^\textrm{out}(t,x) \quad \text{ for } \quad t \gg \epsilon /\pi ^2. \end{aligned}$$

Thus, as implemented in the main text, working solely with the outer solution and neglecting the initial conditions is a rational asymptotic approximation at leading order with respect to $\varepsilon = \Pi _3^{-1} \ll 1$, once initial transients have decayed, that is for times satisfying $t\gg \epsilon /\pi ^2 = \Pi _3^{-1}/\pi ^2$. As the initial transients, and very short times, are not of interest for determining the behaviour of the invasive front of cells for the majority of its propagation we thus work only with the outer equations and solution in the main text.

## Appendix E: Representative Values for Physical and Non-dimensional Parameters

In this Appendix we present order of magnitude parameter estimates for the dynamics of glioblastoma cell cultures in microfluidic devices where chemoattractant gradients play a key role in the progression dynamics, drawing from the theoretical work of Ayensa-Jiménez et al. (2020) and the experimental studies of Ayuso et al. (2017). Table 5 presents characteristic values of the dimensional parameters used to define $\Pi _1, ~\Pi _2, ~\Pi _3, ~\Pi _4$, defined via Eqs. (24a)–(25d), and yielding the order of magnitude estimates

$$\begin{aligned} \Pi _1 = 8.34 \times 10^{-2}, \quad \Pi _2 = 6.67 \times 10^{0}, \quad \Pi _3 = 1.19 \times 10^{3}, \quad \Pi _4 = 7.14 \times 10^{3}. \end{aligned}$$

Note that $\Pi _1\ll 1$ is equivalent to $D\ll 1$ in the original formulation of the non-dimensional model in Sect. 2.2.1.

However, with these parameters the glioblastoma experiments of Ayuso et al. (2017) do not fall into any of the cases considered in the main text, most notably because the equation for oxygen uptake, although approximately time-steady since $\Pi _3,\Pi _4 \gg 1$, reduces to

$$\begin{aligned} v_{xx} = \frac{\Pi _4}{\Pi _3}w_{\text {pc}}(v)u, \quad \frac{\Pi _4}{\Pi _3} \not \ll 1, \end{aligned}$$

which is not considered and more complex than the cases of the main text.

**Table 5:** Parameter estimates. Estimates of dimensional parameters associated with cell culture evolution in microfluidic devices, based on the experimental studies presented by Ayuso et al. (2016, 2017)

Parameter	Value	Units
*D*	$7 \times 10^{-10}$	${\mathrm {cm^2/s}}$
$\chi$	$8 \times 10^{-9}$	${\mathrm {cm^2/mmHg\,s}}$
$\alpha _n$	$1 \times 10^{-6}$	${\textrm{s}}^{-1}$
$c_\textrm{sat}$	$5 \times 10^{7}$	cell/mL
$D_B$	$1 \times 10^{-5}$	${\mathrm {cm^2/s}}$
$\alpha _B$	$1 \times 10^{-9}$	mmHg mL/cell s
$B^*$	$7 \times 10^0$	mmHg
$k_m$	$3 \times 10^0$	mmHg

In terms of the microdevice scenarios considered in Sect. 3.1.2 of the main text it is clear that the above parameters from the microdevice are not consistent with Case (1). This case is associated with sufficiently low cell concentrations to ensure minimal oxygen uptake, requiring the condition $(\Pi _4/\Pi _3) u\ll 1$, which is not consistent with the above order of magnitude parameter estimates. Instead one would require, for instance, cell concentrations, as measured by $c_{\textrm{sat}}$, lowered by two orders of magnitude for example.

Cases (2), (3), respectively require

90 $$\begin{aligned} \Pi _3^{-1}\ll \lambda = \frac{\Pi _4 K}{\Pi _3} \ll \Pi _3, \quad \Pi _3^{-1}\ll \lambda = \frac{\Pi _4 K}{k_m\Pi _3} \ll \Pi _3, \end{aligned}$$

where *K* is the non-dimensional cell density and thus of order unity, and $k_m$ is an non-dimensional version of $k_m$ in Table E, having been non-dimensionalised by $B^*$, and thus also is of order unity. Hence these constraints are satisfied. Thus, the physical scales associated with the microdevice experiments of Ayuso et al. (2017) would be consistent with cases (2) or (3) of Sect. 3.1.2, if surrounding tissue was also present, so as to maintain oxygen consumption in the absence of glioblastoma cells together with either high or low nutrient concentrations. Hence, cases (2) and (3) may be relevant for future microdevice studies.
